# A comparison of 25 complete chloroplast genomes between sister mangrove species *Kandelia obovata* and *Kandelia candel* geographically separated by the South China Sea

**DOI:** 10.3389/fpls.2022.1075353

**Published:** 2023-01-04

**Authors:** Xiuming Xu, Yingjia Shen, Yuchen Zhang, Qianying Li, Wenqing Wang, Luzhen Chen, Guangcheng Chen, Wei Lun Ng, Md Nazrul Islam, Porntep Punnarak, Hailei Zheng, Xueyi Zhu

**Affiliations:** ^1^ Key Laboratory of the Ministry of Education for Coastal and Wetland Ecosystems, College of the Environment and Ecology, Xiamen University, Xiamen, China; ^2^ School of Life Sciences, Xiamen University, Xiamen, China; ^3^ Third Institute of Oceanography, Ministry of Natural Resources, Xiamen, China; ^4^ China-ASEAN College of Marine Sciences, Xiamen University Malaysia, Selangor Darul Ehsan, Malaysia; ^5^ Forestry and Wood Technology Discipline, Khulna University, Khulna, Bangladesh; ^6^ Aquatic Resources Research Institute, Chulalongkorn University, Bangkok, Thailand

**Keywords:** mangrove, *Kandelia*, chloroplast genome, gene diversity, protein dynamics simulation, environment adaptation

## Abstract

In 2003, *Kandelia obovata* was identified as a new mangrove species differentiated from *Kandelia candel*. However, little is known about their chloroplast (cp) genome differences and their possible ecological significance. In this study, 25 whole cp genomes, with seven samples of *K. candel* from Malaysia, Thailand, and Bangladesh and 18 samples of *K. obovata* from China, were sequenced for comparison. The cp genomes of both species encoded 128 genes, namely 83 protein-coding genes, 37 tRNA genes, and eight rRNA genes, but the cp genome size of *K. obovata* was ~2 kb larger than that of *K. candle* due to the presence of more and longer repeat sequences. Of these, tandem repeats and simple sequence repeats exhibited great differences. Principal component analysis based on indels, and phylogenetic tree analyses constructed with homologous protein genes from the single-copy genes, as well as 38 homologous pair genes among 13 mangrove species, gave strong support to the separation of the two species within the *Kandelia* genus. Homologous genes *ndhD* and *atpA* showed intraspecific consistency and interspecific differences. Molecular dynamics simulations of their corresponding proteins, NAD(P)H dehydrogenase chain 4 (NDH-D) and ATP synthase subunit alpha (ATP-A), predicted them to be significantly different in the functions of photosynthetic electron transport and ATP generation in the two species. These results suggest that the energy requirement was a pivotal factor in their adaptation to differential environments geographically separated by the South China Sea. Our results also provide clues for future research on their physiological and molecular adaptation mechanisms to light and temperature.

## 1 Introduction

Mangroves are woody plant communities distributed in tropical and subtropical intertidal zones that play a vital role in reducing the impact of natural disasters and maintaining the coastal ecological environment (Lin, 1999). *Kandelia*, a typical viviparous mangrove species in the Rhizophoraceae family, is widely distributed from Ganges Delta, Burma, through the South China Sea to southern China, and southern Japan (Tomlinson, 1986; Sheue et al., 2003). *Kandelia obovata* (known as *K*. *candel* until 2003) is one of the most important and dominant mangrove species naturally distributed along the coastal areas of southeastern China with the widest distribution and the highest latitude, including in Hainan, Guangdong, Guangxi, and Fujian, in addition to Hong Kong, Macao, and Taiwan (Li & Lee, 1997; Lin, 1999). As the strongest cold-resistant true mangrove species, it is not only a high-quality mangrove species that has been artificially planted for ecological restoration of northward coastal wetlands in China (Liao & Zhang, 2014), such as Yueqing in Zhejiang Province, but also an ideal plant material suitable for exploring the relationship between genetic variation and geographical distribution of mangrove species.

The genus *Kandeli*a originated from Malabar, India, and *Kandelia candel* (L.) Druce was named because of its candle-like hypocotyl propagule. *Kandelia candel* was once regarded as the only species in the *Kandelia* genus (Hou, 1958; Juncosa & Tomlinson, 1988). Comparative analysis of the morphological characteristics of leaves and propagules of *Kandelia* populations collected from Brunei, Thailand, and Hong Kong indicated that distinctive differences exist among them (Maxwell, 1995). Naskar & Mandal (1999) further found that the anatomical structures of the *Kandelia* populations distributed in India and Taiwan were also significantly different. Analysis of the chromosome numbers and karyotypes between populations in the two geographical regions of *Kandelia* showed 2n = 38 for the Indian populations and 2n = 36 for the Japanese populations (Yoshioka et al., 1984; Das et al., 1995). Based on the abovementioned differences in the morphology, structure, and chromosome number, Sheue et al. (2003) proposed that the populations distributed in the south of the South China Sea (including in India, Burma, Thailand, and Malaysia) continue to be recognized as *K*. *candel*, and the populations growing in the north of the South China Sea (including in northern Vietnam, southeastern China, and southern Japan) are recognized as a new species named *K*. *obovata*, after their obovate leaves. Although the *atpB-rbcL* and *trnL-trnF* fragments of the chloroplast (cp) genome were used as molecular markers in the two geographical populations (Chiang et al., 2001), the detailed features of the whole cp genomes between the two population groups and the functions of differential genes in their adaptation to the respective environments need to be further clarified (Takeuchi et al., 2001; Giang et al., 2006; Geng et al., 2008).

The cp genome has the unique advantages of small haploid size, abundant copy number, relatively conservative gene number and arrangement, lack of recombination, and maternal inheritance (Birky, 2001; Rousseau Gueutin et al., 2015). With the development of next-generation DNA sequencing technologies, the complete cp genome has been widely used for plant identification, phylogeny, and evolution studies. Given that cps are important for interactions between a plant species and its environment (including responses to cold, heat, drought, salt, and light), they serve as hubs in cellular reactions to signal and respond *via* retrograde signaling (Bobik & Burch-Smith, 2015). Once there are mutated genes in the cp genome, they might play a pivotal role in the plant’s adaption to a varied environment.

In this study, we employed genome sequencing technology and bioinformatics to conduct a comparative analysis of whole cp genomes between *K*. *candel* samples collected from Malaysia, Thailand, and Bangladesh, and *K*. *obovata* samples collected from the coasts of southeastern China. The results obtained from the present study will advance our understanding of differential adaptation to coastal environments across the world conferred by variations in the cp genomes of closely related mangrove species. On a finer scale, this study will also provide a foundation for further unveiling the differential adaptation mechanisms to light and temperature in *K*. *obovata* and *K*. *candel*.

## 2 Materials and methods

### 2.1 Sampling sites and sample collection

**Figure 1:**
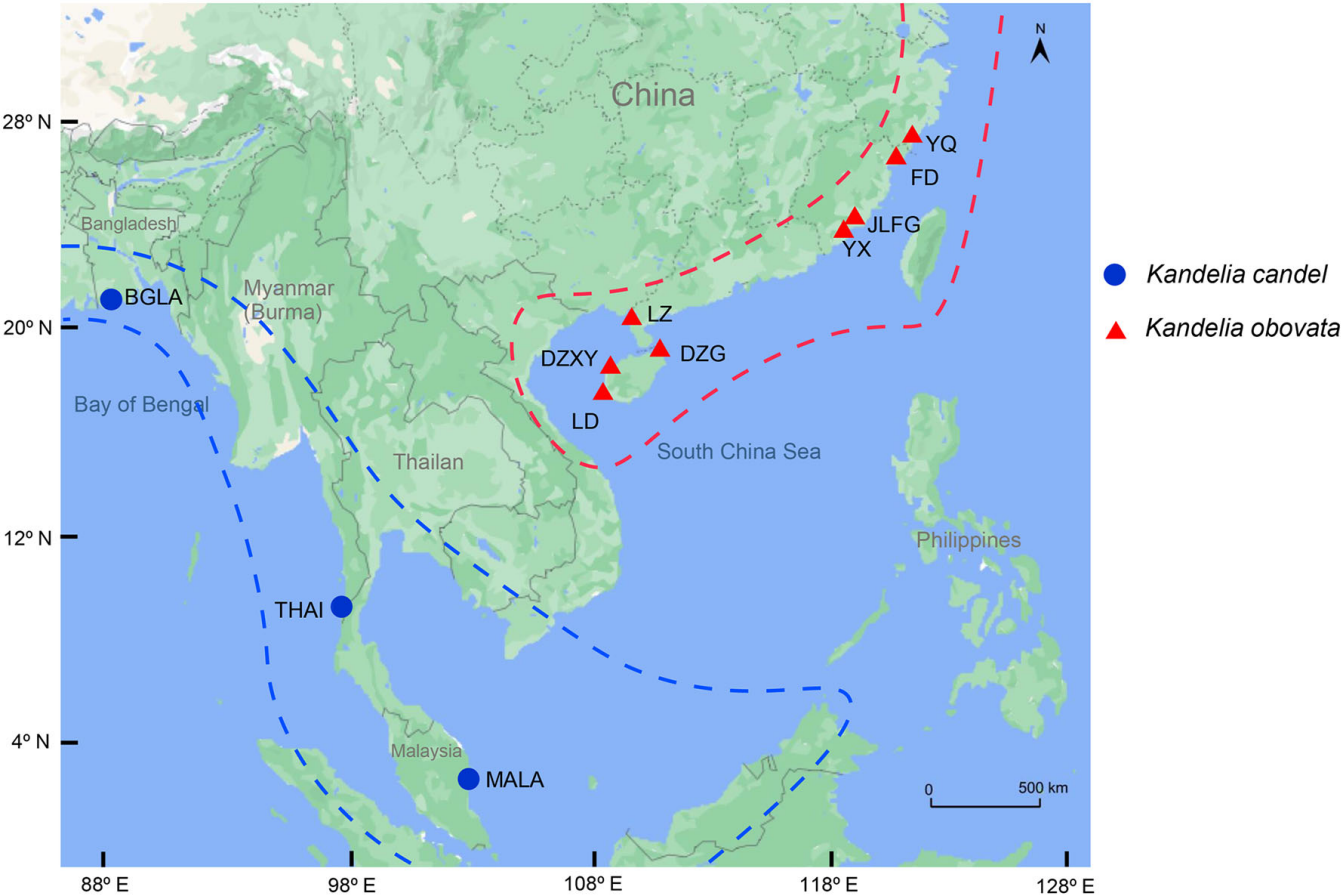
Geographic distribution of 25 *Kandelia* samples collected from 4 different countries including 11 sampling sites for sequencing and assembly the whole chloroplast genomes. The blue solid circle represented *Kandelia candel* species and the red tangle represented *Kandelia obovata* species.

Plant materials of *Kandelia* species were collected from China, Bangladesh, Thailand, and Malaysia, with 18 samples of *K*. *obovata* and seven samples of *K*. *candel*. Samples of *K*. *obovata* were collected from Zhejiang (28°34’N,121°19’E), Fujian (27°29’N, 120°29’E to 23°55’N,117°24’E), Guangdong (19°51’N,110°37’E to 21°37’N,109°47’E), and Hainan (18°45’N,109°10’E to 19°51’N,109°15’E) Provinces in China. Among these, two planted sites, i.e., Yueqing (YQ), Zhejiang Province, and Ledong (LD), Hainan Province, were included. Samples of *K*. *candel* were collected from Khulna, Bangladesh (22°16’N,89°26’E); Ranong, Thailand (10°10’N,98°43’E); and Pahang, Malaysia (3°81’N,103°34’E), which are all located in the west of the South China Sea. Sampling details can be found in **Figure 1** and **Supplementary Table S1**. One to three individual(s) per population were sampled where the population number was 11. The Sampled individuals were at least 10˜15 m apart. Healthy young leaves were collected, dried in silica gel and stored at –20℃ before use. A total of 25 individual samples were used for analysis in this study.

### 2.2 DNA extraction and sequencing

The whole genomic DNA of 25 individual samples was individually extracted from leaf tissues using the cetyltrimethylammonium bromide (CTAB) method (Doyle, 1991). The extracted DNA was dissolved in 60 μL of TE buffer. High-quality DNA (concentration >35 ng/µL) was used in Illumina resequencing. *Kandelia* genome sequencing was performed using the Illumina Hiseq X Ten platform with 150 bp paired-end reads.

### 2.3 Chloroplast genome assembly, gene annotation, and codon usage

Before the *de novo* assembly of the cp genome, quality control of the raw paired-end reads was tested using Trimmomatic v0.40 (Bolger et al., 2014). High quality reads were mapped to cp seed based on bowtie2 v2.3.2 (Langmead & Salzberg, 2012) software with default parameters. The qualitatively assessed paired-end reads from bowtie2 were assembled to produce the cp genome with NOVOPlasty v4.3.1 software (Dierckxsens et al., 2020) using the published cp genome of *Kandelia obovata* (GenBank Acc. No. MN117072) as a reference (Yang et al., 2019). GetOrganelle software (Jin et al., 2020) was also applied to complete and double-check the assembly of the cp genomes. The coverage of reads in the nuclear genome was measured, which used passed Trimmomatic reads mapping to published nuclear genome based on bowtie2 v2.3.2 (Langmead & Salzberg, 2012) and the mapped genome reads were counted to coverage depth. We also used the same methods to employed the coverage depth of *Kandelia* cp genoma. The complete cp genome was annotated and corrected using Geseq (Tillich et al., 2017) and CPGAVAS2 (Shi et al., 2019). The circular gene map was visualized using OGDRAW (Greiner et al., 2019). In order to ensure the reliability and accuracy of assemble and annotation results, we have carried out manual comparing our results with published cp genomes of *K. obovata* (Yang et al., 2019a and 2019b). Two genes, *psbI* and *psbK*, were absent in the previously published cp genome of *K. obovata* (GenBank Acc. No NC042718, MN313722), but they were located in the cp genomes of the mangrove Rhizophoraceae family, such as in *Rhizophora apiculata* (GenBank Acc. No. MT129631.1) and non-Rhizophoraceae families, such as *Avicennia marina* in the Acanthaceae family as shown in our previous research (GenBank accession number: MT108381), as well as in our present study. Therefore, we designed primers around gene *psbK* and performed PCR amplification of cp genomes for further confirmation. The sequence of ATATTTGAATTTGAATTGAGTTTCGGT was used as *psbK*-around-F primers. The sequence of GGTTTGTTGGATGTGCTGTGA was used as *psbK*-around-R primers. The annotated chloroplast genome sequences for the 25 samples of *Kandelia* have been deposited in the GenBank database under accession numbers successively from ON969308 to ON969332 (**Supplementary Table S1**). To identify codon usage patterns, all coding sequences (CDS) were subsequently used for the estimation of relative synonymous codon usage (RSCU) through the CUSP program with EMBOSS v6.5.7 with default parameters (Rice et al., 2000).

### 2.4 SNP calling, PCA, and phylogenetic tree

**Figure 2:**
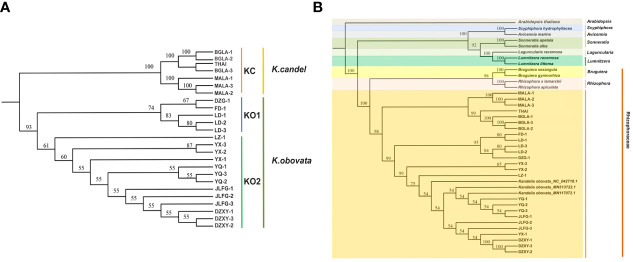
Phylogenetic tree. **(A)** The *Kandelia* tree constructed using the UPGMA model based on the 76 single-copy genes in the 25 whole cp genomes. **(B)** The tree constructed using the UPGMA model based on 38 homologous pair genes in 13 mangrove species including 40 samples.

Chloroplast genome sequences of the 25 individuals from both *K. candel* and *K. obovata* were comparatively analyzed using BWA v0.7.12 with default parameters (Li & Durbin, 2009), which were aligned to the longest cp genome of *K. obovata* (YQ-2). Single-sample variant calling was performed with the Genome Analysis Toolkit (gatk v4.2.2.0) (McKenna et al., 2010) with default parameters. Gatk SortSam, gatk MarkDuplicates, gatk HaplotypeCaller and gatk CombineGVCFs were combined for SNPs calling. High-quality SNPs were kept and filtered using vcftools software (Danecek et al., 2011) with the following parameters: max missing of 0.6 and minQ of 30. The EIGENSOFT v7.2.1 package (https://github.com/gurinovich/PopCluster) was used to perform PCA, and EIGENSTRAT (Alexander et al., 2009) was performed on linkage disequilibrium (LD)-pruned pseudomolecule SNPs. The p-distance matrix was calculated using VCF2Dis (v1.47) (https://github.com/BGI-shenzhen/VCF2Dis) with the filtered SNP set from the 25 *Kandelia* accessions. A neighbor-joining tree was reconstructed using the UPGMA method, and MEGA (v5.2) (Kumar et al., 2018) was used to visualize the tree. To determine the phylogenetic relationship within the genus *Kandelia*, complete cp genomes were compared with the 25 samples among 76 shared single copy protein-coding genes to build a phylogenetic tree. The 76 genes were explored by ORTHOMCL v6.11 (Chen et al., 2006). The amino acid sequence alignments were based on MAFFT v7.487 (Katoh & Standley, 2013) with default parameters. The phylogenetic trees of *K. candel* and *K. obovata* were established with the UPGMA method by utilizing 76 shared single-copy genes within amino acid sequence (**Figure 2A**) based on MEGA (v5.2) (Kumar et al., 2018). To understand the phylogenetic position of the two well-differentiated geographical sets of *Kandelia* in mangroves, 13 published cp genomes of seven mangrove genera (*Rhizophora, Bruguiera, Lumnitzera, Laguncularia, Sonnerati, Avicennia*, and *Scyphiphora*) were downloaded from NCBI. *Arabidopsis thaliana* (NC_000932.1) (Sato et al., 1999) was used as the outgroup. ORTHOMCL v6.11 (Chen et al., 2006) was applied to identify orthologous gene families in 40 cp genomes. Single-copy orthologues were identified, and the BLASTP E-value was below 10e^-5^. Using 38 cp single-copy protein-coding genes (*accD*, *atpB*, *atpE*, *atpF*, *atpH*, *atpI*, *ccsA*, *cemA*, *matK*, *ndhA*, *ndhC*, *ndhD*, *ndhE*, *ndhG*, *ndhH*, *ndhI*, *ndhJ*, *ndhK*, *petB*, *petD*, *petG*, *petL*, *petN*, *psaA*, *psaB*, *psaC*, *psaJ*, *psbB*, *psbC*, *psbD*, *psbE*, *psbH*, *psbJ*, *psbL*, *psbM*, *psbN*, *psbT* and *rbcL*), a phylogenetic tree was reconstructed by amino acid sequence. Multiple sequence alignments of shared gene datasets were generated with MAFFT v7.487 (Katoh & Standley, 2013) with default parameters. The UPGMA method was used to infer the phylogenetic tree using MEGA (v5.2) (Kumar et al., 2018), and the parameters were adjusted for the tree with 1000 bootstrap replicates using the JTT matrix-based method with the units of the number of amino acid substitutions per site.

### 2.5 Comparative chloroplast genome structure analyses

Tandem repetitive sequences were determined using the online program Tandem Repeats Finder (v 4.09) (Benson, 1999) according to the following criteria: match value of 2, mismatch value of 7, delta value of 7, match probability of 85, indel probability of 10, minscore value of 50, and maxperiod value of 500. The tuple sizes were 0, 4, 5, and 7, and the tuple distances were set to 0, 29, 159, and 500. The program REPuter (Kurtz et al., 2001) was used to identify and determine the locations and sizes of forward, reverse, palindrome, and complement sequences having the following parameters: minimum of 30 bp, sequence identity greater than 90%, and maximum computed repeats of 4500. A perl program MicroSAtellite (MISA v2.1) (Beier et al., 2017) identification tool was used to detect simple sequence repeats (SSRs) in the 25 cp genomes. In this study, only perfect repeats were selected for analysis with the following parameters: basic motifs (1~6 bp) and a minimum repeat length of 8 bp (for mono- and di-), 12 bp (for tri- and tetra-), 15 bp (for penta-), 18 bp (for hexa-), and the minimum distance between two SSRs was set to 100. The variations between *K. candel* and *K. obovata* were identified by Genome Varscan (parameters: Diff OneInHundred, VarRange 1-100) and then the primers were designed by Batch Target Region Primer Design with default parameters, the above two tools were used under TBtools v1.099 (Chen et al., 2018) software.

To determine the sequence divergence of the *Kandelia* cp genomes among the 25 samples, the online genome comparison tool mVISTA (https://genome.lbl.gov/vista/index.shtml) was used with the *K. obovata* (YQ-2) annotation as the reference. The default parameters were set to align the cp genome in Shufe-LAGAN mode, and the sequence conservation profile was visualized using an mVISTA plot. Based on the comparative genome size results, *K. candel* (MALA-2) and *K. obovata* (YQ-2) were selected to visualize the cp genome gene order and the collinear blocks between the two species under *Kandelia*. The comparison was performed using Mauve v2.3.1 (Darling et al., 2004) with default iterative alignment, seed weight, sum of pairs LCB scoring, and LCB settings. DnaSP v5.10 (Rozas et al., 2017) was applied to determine the level of nucleotide diversity (*Pi*) among 25 samples, with the *K. obovata* (YQ-2) cp genome as the reference. When DnaSP6 calculated the *Pi* value, the step size was set to 650 bp, and the slide window size was 650 bp. The intraspecific *Pi* values of *K. candel* and *K. obovata* were also calculated using these methods. We extended the region with high *pi* value (*Pi >*0.01) up and down 1000bp to find the gene closest to this region for further analysis. The ratio of the number of non-synonymous (Ka) substitutions to the number of synonymous (Ks) substitutions (Ka/Ks) of each protein-coding gene was estimated using perl script ParaAT2.0 (Zhang et al., 2012b) with muscle v3.8.31 (Edgar, 2004) and KaKs_Calculator2.0 (parameters: -c 11 -m MS) (Wang et al., 2010).

### 2.6 Structural modeling and molecular dynamics simulation of *ndhD* and *atpA*

Each pair of genes of *Kandelia* was aligned using CLC Main Workbench 6 software (https://digitalinsights.qiagen.com/). Loci with variations in the species-specific genes with non-synonymous mutations were the focus of our attention between *K. candel* and *K. obovata*. We searched the genes with mutations and identified mutated sites located in the domain area with SMART (Letunic et al., 2021). The *ndhD* and *atpA* genes met the criteria. The structural models of *ndhD* and *atpA* were then built.

Templates for homologous modeling were searched *via* BLASTP (Johnson et al., 2008) on the Protein Data Bank (PDB) database (Burley et al., 2021) with a criterion of sequence identity > 30% (more is better). With a comprehensive consideration of E-value and query coverage, an optimal template was selected by PDB ID 1FX0_A (Groth & Pohl, 2001) for *atpA* and 6HUM_D for *ndhD* (Schuller et al., 2019). Model building for *K. candel* proteins was then carried out on MOE software (https://www.chemcomp.com/Products.htm) with default settings. *K. obovata* proteins were obtained by modifying the *K. candel* model with corresponding mutation positions. MOE Protein Builder was used for specific amino acid mutation followed by an energy minimization choice of “Selected Sidechain + Tether BB”.

Molecular dynamics (MD) simulations were performed using the GROMACS v2021.5 (Van Der Spoel et al., 2005) with GROMOS 54A7 force field (Nathan et al., 2011). Systems were solvated with a water model (spc216.gro file) in a dodecahedron box with a boundary distance of 1.0 nm to the box edge. To maintain a neutral condition, counter-ions were added to the systems (6 Na^+^ for *atpA*, 2 Cl^-^ for *ndhD*). The initial structures were then optimized by an energy minimization process using the Steepest Descent method until the energy gradient was ≤ 10 kJ mol^-1^ nm^-1^. Subsequently, a two-stage equilibration process was conducted. The first 100-ps NVT equilibration for temperature control and the second 100-ps NPT equilibration for pressure control. The final MD simulations lasted 50 ns at a constant temperature of 300 K and a constant pressure of 1 atm for each system. Root Mean Square Deviation (RMSD), root-mean-square fluctuations (RMSF), and b-factor value of the residues were calculated from the MD trajectory files using the embedded commands in GROMACS. Pymol v2.4.1 (https://github.com/schrodinger/pymol-open-source) was used for protein structure visualization and mapping the b-factor value to the corresponding residue on the graph. As the b-factor value went from small to large, the colors changed in the following order: grey - blue -yellow - orange - red.

To study the interaction between ATP-A and ADP, molecular docking was carried out on MOE software with a General docking scenario. The potential binding site was selected based on the result of the Site Finder application in MOE.

To explore the consistency of ATP-A and NDH-D sequences changes and genes differentiation in mangrove plants and model angiosperms *Arabidopsis thaliana.* The proteins sequence of APA-A and NDH-D of six different species were compared and visualized with CLC Main Workbench 6 software (https://digitalinsights.qiagen.com/). Based on the comparison results, the phylogenetic trees of these two proteins were established respectively, the parameters as follows: Algorithm NJ, replicates 1000.

## 3 Results

### 3.1 General features of *Kandelia* complete chloroplast genomes

**Figure 3:**
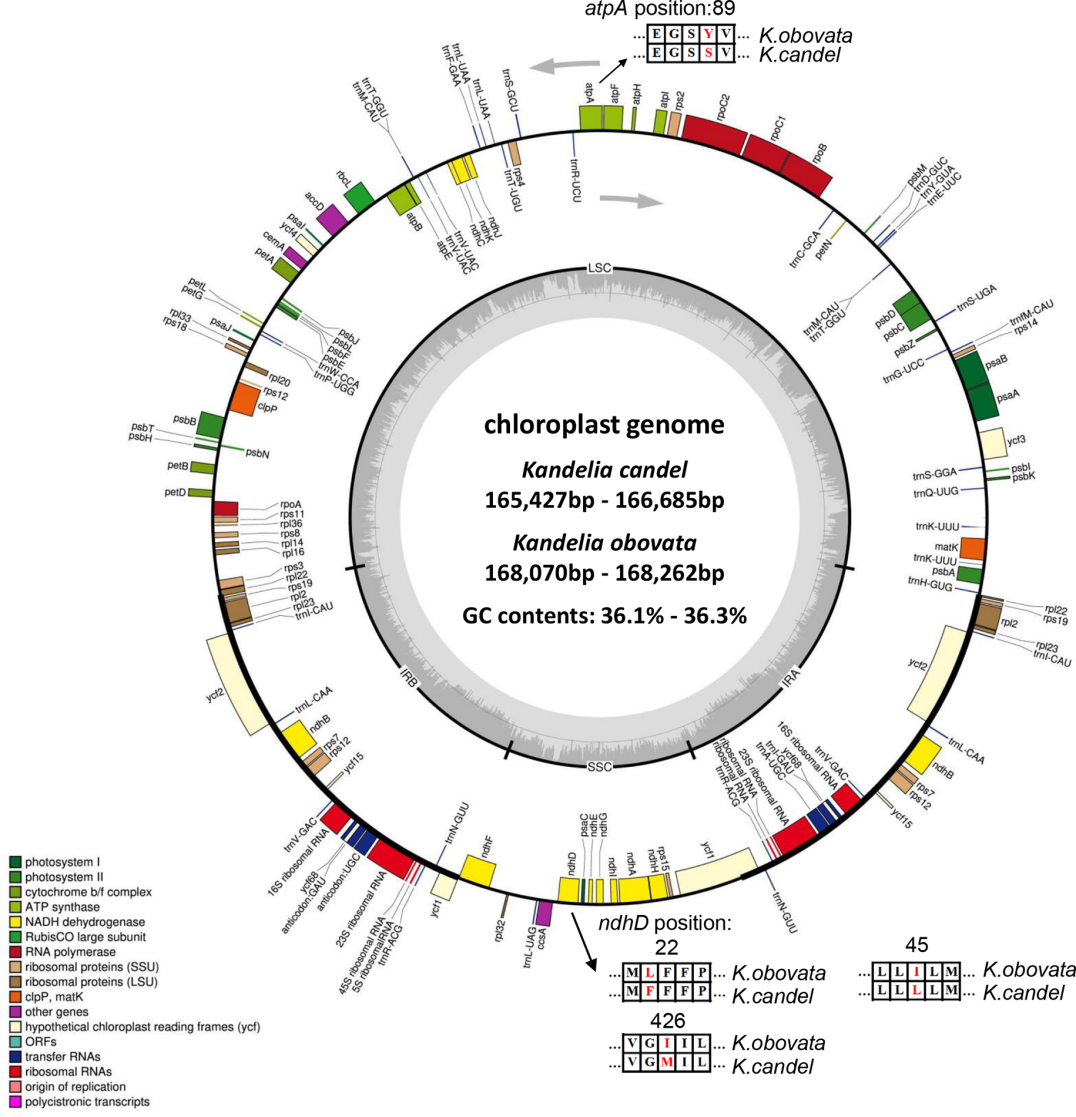
Gene maps of the chloroplast genomes of *Kandelia*. Genes shown outside the outer circle are transcribed clockwise, and genes shown inside the circle are transcribed counterclockwise. Genes belonging to different functional groups are color coded. The dashed area in the inner circle indicates the GC content of the chloroplast, and the light gray area corresponds to AT content of the chloroplast. Point mutations of *atpA* and *ndpD* gene were shown between *K. obovata* and *K. candel*.

A total of 25 samples of *Kandelia* were used to obtain 5~10 Gb raw reads with a mean coverage of ~25× to 50× of whole genomes and 2298× to 3484× of cp genome base coverage. The amplified sequences were consistent with the assembly results, which illustrated that the *psbI* and *psbK* genes were present in *Kandelia* cp genomes (**Supplementary Figure S1**), and the assembly of the cp genomes was correct (**Figure 3**). The circular maps of the 25 cp genomes are shown in **Figure 3**. The 25 cp genomes ranged from 165,247 to 168,262 bp in length. The total sizes of *K. candel* ranged from 165,247 to 166,729 bp, while the sizes of *K. obovata* ranged from 168,070 to 168,262 bp. The length of *K. obovata* was approximately 2 kb longer than that of *K. candel* on average. All cp genomes had a circular assembly with a typical quadripartite structure, which was composed of large and small single-copy (LSC and SSC) regions and two inverted repeats (IRs) (**Figure 3**, **Table 1**). The LSC length showed the greatest difference between the two species, being 92,380~93,683 bp for *K. candel* and 94,711~94,908 bp for *K. obovata*, which resulted in the differential length of the two species. GC contents in both LSC (38.2~8.3%) and SSC (28.1~28.6%) were slightly lower in *K. obovata* (**Supplementary Table S4**).

**Table 1:** The basic chloroplast genome information of the 25 *Kandelia* accessions.

Characteristics	*K. candel*	*K. obovata*
Total Number of Raw reads	7,183,966,500 - 8,404,770,000	5,793,072,000 - 10,367,439,000
Total Number of Mapped read	2,899,354 - 3,771,743	2,605,052 - 3,949,533
Percent of chloroplast genome reads (%)	0.04% - 0.045%	0.038% - 0.045%
Chloroplast genome coverage (X)	2,558 - 3,328	2,298 - 3,484
Total size (bp)	165,247- 166,729	168,070 - 168,262
LSC length (bp)	92,380 - 93,683	94,711 - 94,908
IR length (bp)	266,25 - 266,87	266,47 - 266,72
SSC length (bp)	196,70 - 199,25	199,64 - 200,39
Total genes	128	128
Protein coding genes	83	83
tRNA genes	37	37
rRNA genes	8	8
Overall GC content (%)	36.10 - 36.30	36.1
GC content in LSC (%)	38.3	38.2
GC content in IR (%)	42	42
GC content in SSC (%)	28.10 - 28.60	28.10 - 28.20
Accession number	ON969322 - ON969325, ON969308,ON96939,ON969332	ON969310 - ON969321, ON969326 - ON969331

The LSC region comprised 59 protein-coding genes and 22 tRNA genes, and the SSC region comprised 11 protein-coding genes and one tRNA gene in the 25 *Kandelia* cp genomes. Eighteen genes contained introns, of which 15 genes (*trnK-*UUU, *rpoC1*, *atpF*, *trnG-*UCC, *trnL-*UAA, *trnV-*UAC, *petB*, *petD*, *rpl2*, *ndhB*, *trnI-*GAU, *ndhA*, *trnQ-*UUG, *trnS-*GGA and *trnA-*UGC) contained one intron, while two genes (*ycf3* and *clpP*) possessed two introns (**Supplementary Table S2**). The *trnk-*UUU gene featured the longest intron (2595~2607 bp), which contained the *matK* gene. The shortest intron was located in *trnL-*UAA (563~569 bp). The *rps12* gene was a trans-spliced gene with the 5’-end situated in the LSC region, with one exon, and the 3’-end situated in the IR region.

### 3.2 Codon usage analysis

The ATT codon was the most abundant in the *Kandelia* cp genomes (42.87%). The TGA codon was the least used in *Kandelia* cp genomes (2.69%). The analysis of relative synonymous codon usage (RSCU) values revealed the dominance of 20 amino acids. RSCU values were computed for *K. candel* and *K. obovata* cp genomes based on their protein-coding sequences. **Supplementary Figure S2** shows the codon content of 20 amino acids and stop codons in all protein-coding genes in the cp genomes of the two species. The coding regions of *K. candel* were composed of 24,542, 24,680, and 24,690 codons. In *K. obovata*, the coding regions were composed of 24,674, 24,681, and 24,680 codons. The most prevalent amino acid was leucine, with 2,579~2,592 codons in *K. candel* and 2591 codons in *K. obovata*, while the rarest was cysteine, with 306 codons in *K. candel* and 307 codons in *K. obovata*, showing the sequence diversity between the two species. When codons with no preference value were set to 1.00, codons for leucine, serine, and arginine were the most abundant (RSCU = 6), while those for tryptophan and methionine were the least abundant (RSCU = 1) (**Supplementary Figure S2**). In addition, nearly all A/T-ending codons had RSCU values >1, whereas G/C-ending codons had RSCU values <1.

### 3.3 SNPs analysis and construction phylogenetic trees

**Figure 4:**
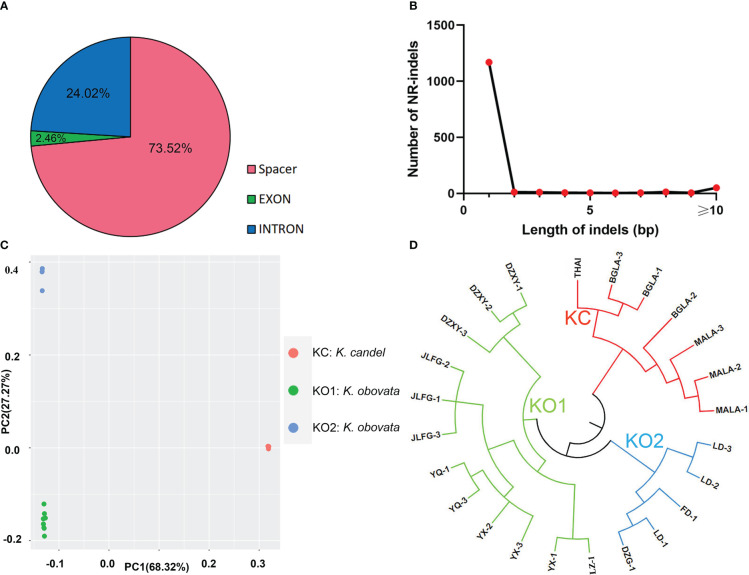
Analyses of indels in the chloroplast genomes. **(A)** Frequency of different indels types and locations. **(B)** Number and size of non-SSR-related indels in the *K*. *obovata* genomes. **(C)** The PCA result of SNPs. **(D)** The phylogenetic tree based on SNPs data.

There were 1522 indels in the 25 cp genomes of *Kandelia*, comprising 112 (7.36%) SSR-related indels and 1410 (92.64%) non-SSR-related indels. In total, 73.52% of the indels were present in 1141 intergenic space regions, while 2.46% of the indels were located in exons and 24.02% were present in the introns (**Figure 4A**). The *atpE*-*atpB*-*rbcL* regions contained 60 indels, followed by *trnS* (GCU)-*rps4*-*trnT* (UGU) with 44 indels and *trnD* (GUC)-*psbM*-*petN* with 33 indels. The single nucleotide site generally displayed a high frequency of SSR-related indels in the present study; however, we also found five indels located in the *trnS* (GCU)-*rps4*-*trnT* (UGU) region, which were 13~56 bp in length. All SSR-related indels were A/T-type SSRs. Three SSR-related indels were located in the coding regions, and the other 41 SSR-related indels were found in the non-coding regions. The sizes of the non-SSR-related indels ranged from 1 to 169 bp, with one bp indel (1,171) being the most common (**Figure 4B**). The largest indel (88 bp) in the spacer of *trnK* (UUU)–*trnQ* (UUG) was a deletion in *K. candel*. The second largest indel was in the spacer of *ndhG*–*ndhI* (81 bp), which was also a deletion in *K. candel*.

The relationships among the 25 samples were analyzed using principal component analysis (PCA) based on SNPs data, and a phylogenetic tree of the SNP markers in the whole cp genome was identified (**Figures 4C, D**). Using the longest cp genome of YQ-2 as the reference genome, the other 24 cp genomes were clustered into two main groups, KC and KO, the latter included KO1 and KO2 subgroups. The KC group belonged to *K. candel* and included all samples (THAI, BGLA-1, BGLA-2, BGLA-3, MALA-1, MALA-2, and MALA-3) of *K. candel*. The KO1 and KO2 groups belonged to *K. obovata*, which consisted of DZXY1-3, JLFG1-3, YX1-3, YQ-1, YQ-3, and LZ-1 in the KO1 group and LD-1, LD-2, LD-3, FD-1, and DZG-1 in the KO2 group. The first two principal components comprised 68.32% of the total variance, with PC1 reflecting the variability of the *K. candel* and *K. obovata* groups.

The phylogenetic trees of *K. candel* and *K. obovata* within the genus *Kandelia* were established with the UPGMA method by utilizing 76 shared single-copy protein genes (**Figure 2A**). The phylogenetic trees showed that the individuals of *K. candel* clustered in one clade as a KC group. *Kandelia obovata* individuals were gathered in another clade, and samples were gathered further into the KO1 and KO2 subgroups, which was consistent with the phylogenetic tree constructed based on SNPs (**Figure 4D**). The KC branch of *K. candel* was subdivided into two clades: MALA1-3 for one clade and BGLA1-3 together with THAI for another clade. The KO1 and KO2 groups of *K. obovata* branches showed that most samples from the same location were more closely related; however, some samples collected from neighboring provinces or even far from each other also showed closer kinship. For example, LD 1-3 and DZG-1, both from Hainan Province, were clustered with FD-1 from Fujian Province, and YQ 1-3 from Zhejiang Province was grouped with JLFG-1 from Fujian Province. This might be because Fujian Province was one of the major collection areas for propagules of *K. obovata* in artificial introductions or transplants.

In addition, a phylogenetic tree based on 38 homologous pair genes was constructed among 7 genera, 13 mangrove species, including 39 samples (**Figure 2B**). The results illustrated that *Kandelia* was closely related to *Bruguiera* and *Rhizophora* under the Rhizophoraceae family, but they were well differentiated into two species. In terms of the 25 *Kandelia* samples, this result also coincided with the phylogenetic trees constructed with SNPs (**Figure 4D**) and 76 shared single-copy genes in these cp genomes (**Figure 2B**), all illustrating that the genus *Kandelia* consisted of two distinct species.

### 3.4 Comparative analysis of cps genomes structural variations

The distribution of long repeats in *Kandelia* cp genomic sequences was analyzed and summarized, as shown in **Supplementary Figure S3A**. There were three types of repeats: tandem repeats (TRs), forward repeats (FRs), and palindromic repeats (PRs) (**Supplementary Figure S3A**). By searching the repeats of each of the 25 cp genomes, the number of repeats was significantly different in TRs and PRs between *K. candel* and *K. obovata*. There were 2~10 TRs in *K. candel* and 17~22 TRs in *K. obovata* (**Supplementary Figure S3B**). The average number of TRs in *K. candel* was 11, which was significantly lower than that in *K. obovata* (7). In terms of FRs, the mean number was 37 in the *K. candel* cp genomes, whereas it was 59 in *K. obovata* genomes. In contrast, the PR numbers in *K. candel* were greater than those in *K. obovata*, at 5~8 and 2~4, respectively. The highest number of repeats (88) was found in the LD-3 cp genome of *K. obovata*, with 19 TRs, 66 FRs, and three PRs. Among these repeats, 47 of the forward repeats were found with 28~46 bp, six repeats with 47~64 bp, five repeats with 65~83 bp, four repeats with 84~102 bp, three repeats with 103~120 bp, and one repeat with length longer than 121 bp (**Supplementary Figure S3C**). The lowest number of repeats (a total of 41) was found in the BGLA-1 cp genome of *K. candel* (nine TRs, 27 FRs, and five PRs), of which 26 of the FRs were found with 28~46 bp, 14 repeats had length lower than the average value (40) of *K. obovata*, and one repeat had a length of 47~64 bp (**Supplementary Figure S3A, C, D**).

The distribution of three types of SSRs, namely mononucleotides, dinucleotides, and trinucleotides, is shown in **Supplementary Figure S4A**. The total number of SSRs were 73 to 78 with an average density from 456.25 SSRs/Mb to 487.5 SSRs/Mb of *K. obovata*. In *K. candel*, the SSRs density were from 456.25 SSRs/Mb to 506.25 SSRs/Mb (73~81). AAT repeats were found in *Kandelia*, and there was only one trinucleotide type duplication per cp genome. In addition, the SSR sequences of the whole cp genome displayed a prevalence of AT-rich mononucleotides (98~100%) and dinucleotides (100%).

The mononucleotide SSR was found to be the most abundant, followed by dinucleotides and then trinucleotides, in both species. They accounted for 91.86%, 6.82%, and 1.32% of total SSRs in *K. obovata* individuals, on average, while they accounted for 92.33%, 6.37%, and 1.30% in *K. candel* individuals (**Supplementary Table S3**). Most of the SSRs in the 25 cp genomes were found in the LSC region (**Supplementary Figure S4B**). A significant difference in SSR distribution between *K. candel* and *K. obovata* was also found in the LSC region. The number of SSRs in the LSC region was 53 (YX-1) to 61 (MALA-1, 2, and 3). The average SSR number in *K. candel* (59) was greater than that in *K. obovata* (56). The most mononucleotide SSRs distributed in the LSC region in *K. candel* were 54, on average, whereas there were 50 in *K. obovata*.

Similarly, SSRs in the SSC region were mostly mononucleotides, but SSRs as dinucleotides were also found in *K. candel* individuals. The number of SSRs in the SSC region was 9~12 (average of 14.37%) in *K. obovata*, which is higher than that in *K. candel* (i.e., 8~11, average of 11.99%). In the IRa region, four to six mononucleotide SSRs were found. The least SSRs were found in the IRb region with four mononucleotides, except MALA-2 with five mononucleotides. The DZXY cp genome of *K. obovata* had one dinucleotide SSR in the IRb region, which was not found in any of the other samples. Molecular markers to distinguish these two species were shown as in **Supplementary Table S5**.

**Figure 5:**
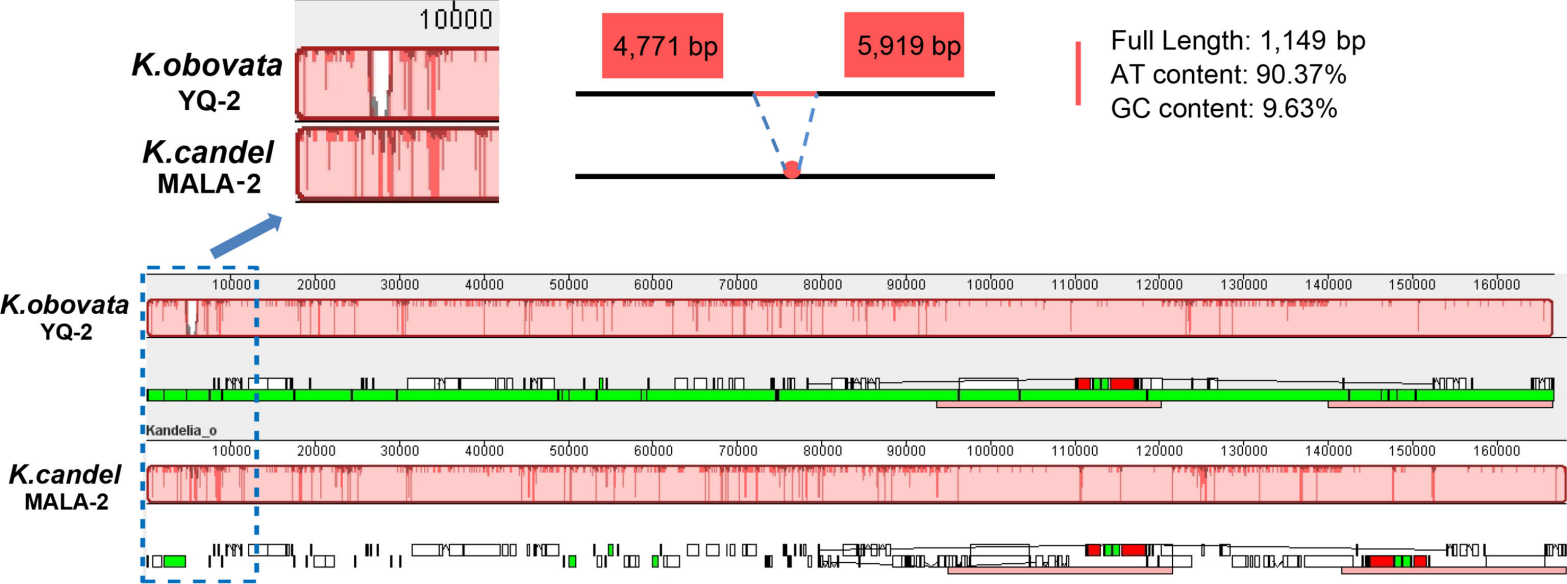
Gene map comparison between *K.candel* and *K.obovata* chloroplast genomes aligned using Mauve, showing a big ‘gap’ of 1,149bp with rich A and T in LSC regions in *K. obovata*.

Using the longest *K. obovata* (YQ-2) cp genome as the reference, the comparative sequence analyses exhibited high sequence similarities and high gene structure order consistency among the 25 cp genomes (**Supplementary Figure S5**). The co-linearity in gene placement between the cp genomes of *K. candel* and *K. obovata* was analyzed with MALA-2 and YQ-2 as representatives. The results indicated that gene clusters had no changes, whether in the single-copy regions or in inverted repeat regions between the two species, showing high conservation at the whole cp genome level (**Figure 5**). However, a gap of 1,149 bp in YQ-2 was detected in the LSC region (**Figure 5**). The gap region was very rich in AT (90.37%).

**Figure 6:**
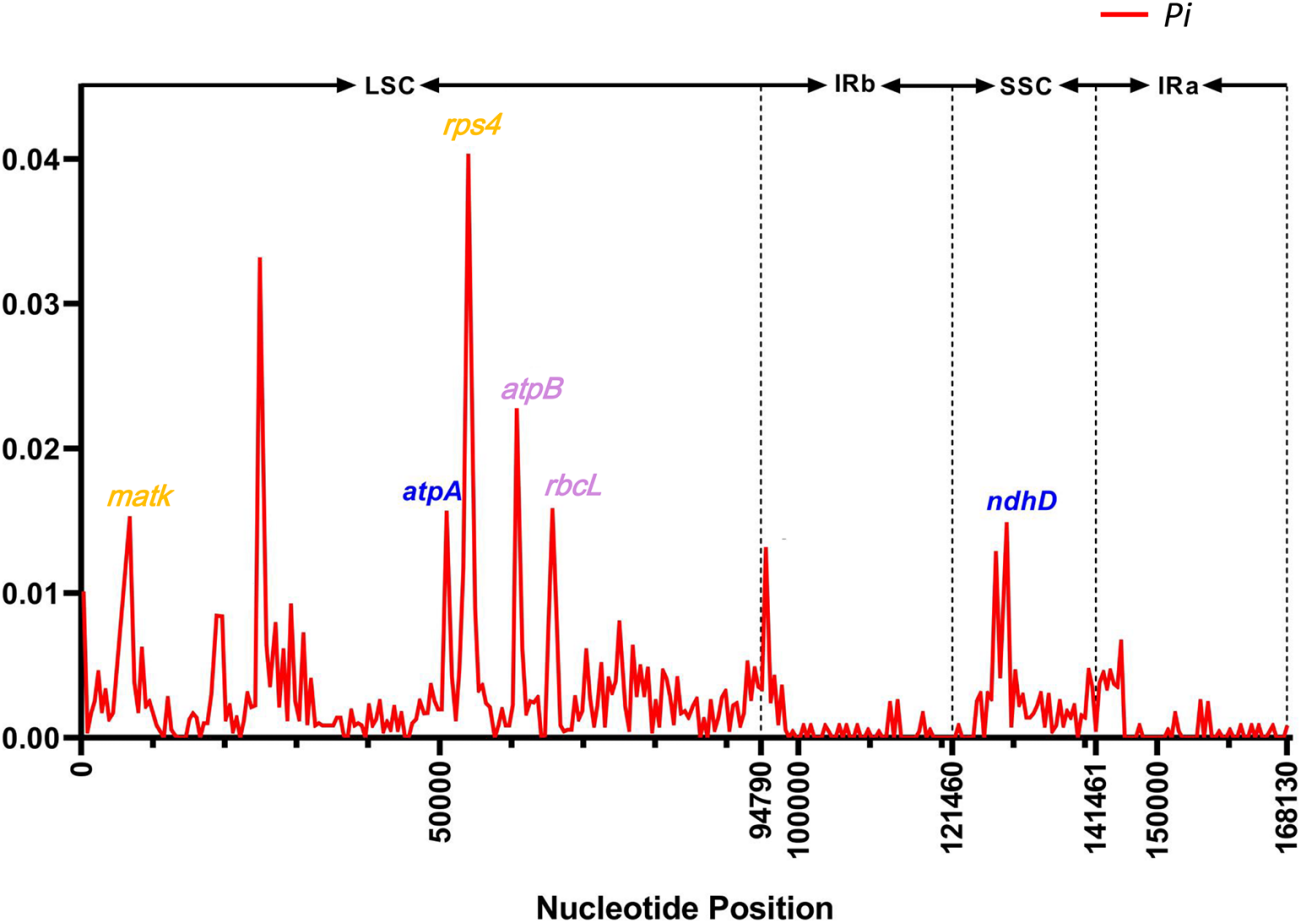
The *Pi* value in sliding-window analysis of the whole chloroplast genomes. The genes highlighted in blue color showcase the SNPs between *K. candel* and *K. obovata*. The genes highlighted in orange color were SNPs within *K. candel* species. The purple color highlighted genes were SNPs within *K. obovata* species.

The nucleotide diversity (*Pi*) value was calculated using the DnaSP program to evaluate the mutation hotspots in the 25 cp genomes (**Figure 6**). The results illustrated that the Pi values varied from 0 to 0.04 in the peer window of the 25 *Kandelia* cp genomes. Seven of these loci, i.e., *atpA* (0.0156), *ndhD* (0.0148), *matK* (0.0152), *rps4* (0.0402), *atpB* (0.0228), and *rbcL* (0.0157), were located at high values (*Pi >*0.01) area. Furthermore, sequence coherence analysis showed that the *atpA* gene in the LSC region and the *ndhD* gene in the SSC region displayed two distinct patterns between *K. candel* and *K. obovata*, which aroused our interest in further comparison. In addition, the *matK* gene and the *rps4* gene in the LSC region displayed a consistent pattern in all samples of *K. candel*. The *atpB* gene and the *rbcL* gene in the LSC region displayed a consistent pattern in all samples of *K. obovata*. The *Pi* value of intraspecific cp genome nucleotide diversity in *K. candel* was between 0 and 0.023, while it was 0~0.015 in *K. obovata*. The Ka and Ks values estimated for each protein-coding gene showed that they were in the range of 0 to 0.5, and none of the Ka/Ks values for these genes were greater than 1 in the present study. In particular, the Ka/Ks value of *atpA* gene is 0.10 and that of *ndhD* gene is 0.26. (**Supplementary Figure S6**, **Supplementary Table S3**).

### 3.5 Structure analysis and molecular dynamics simulation of NDH-D and ATP-A proteins

Analysis of the SNPs between *K. candel* and *K. obovata* showed that the *ndhD* and *atpA* genes had some SNPs located in highly variable regions (**Figure 6**). All the mutation sites of proteins NDH-D and ATP-A were identified in the domain area using the SMART program (**Supplementary Figures S7A, S8A**). To explore the influences of mutation sites of NDH-D and ATP-A on protein structures, we carried out homology modeling, molecular docking, and a followed-up molecular dynamic (MD) simulation. b-factors (**Supplementary Figure S7B**), RMSD (**Supplementary Figure S8B**), and RMS fluctuation (**Supplementary Figure S8C**) were calculated for better understanding.

**Figure 7:**
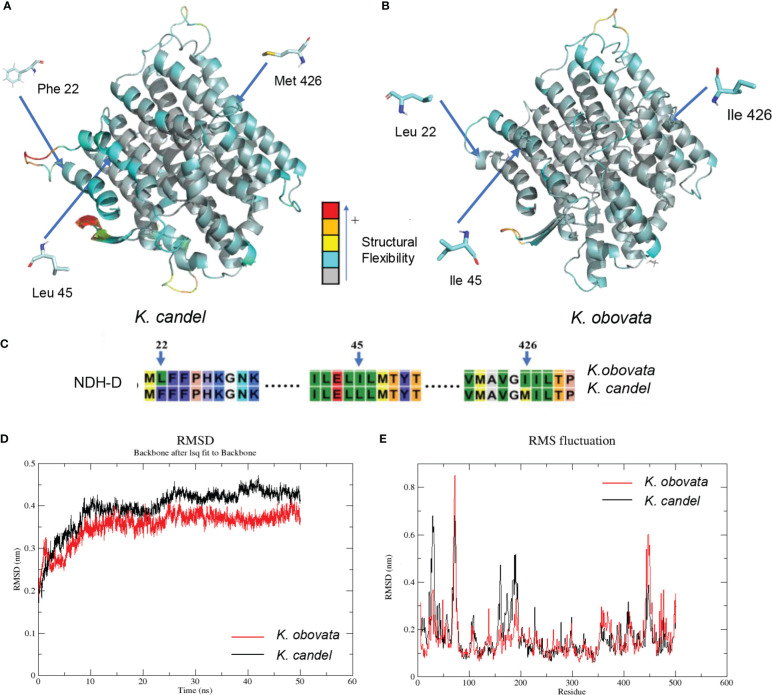
The predicated 3D structural modeling and molecular dimnamics simulation of the NDH-D protein. By setting different colors (gray to blue to yellow to orange to red) according to the b-factor values, the color in the structure is closer to red, the more flexible the structure. **(A)** The 3D model of NDH-D protein for *K*. *candel*. **(B)** The 3D model of NDH-D protein for *K*. *obovata*. **(C)** The mutation sites of protein sequence compared between *K*. *candel* and *K*. *obovata*. Molecular dynamics simulations showed with RMSD **(D)** and RMS fluctuation **(E)**.

The NDH complex D sub-protein (NDH-D) was reconstructed with the *K. candel* protein model by selecting the appropriate homologous protein, and *K. obovata* proteins were obtained by modifying the *K. candel* NDH-D protein model with corresponding mutation positions (**Figure 7**). As shown in **Figure 7C**, Phe22, Leu45, and Met426 of NDH complex D sub-protein (NDH-D) in *K. candel* were substituted by Leu22, Ile45, and Ile 426 in *K. obovata*. Clearly, due to the sense mutations of the protein amino acids in *K. obovata*, the protein structure changed accordingly (**Figures 7A, B**). After MD simulations, b-factors for residues 22, 45, and 426 were calculated. The b-factors for the 22nd, 45th, and 426th sites were all higher in *K. candel* than in *K. obovata* (**Supplementary Figure S7B**), indicating a more flexible conformation at these three sites in *K. candel*. Although the overall trend of the conformational changes of NDH-D in both *K. candel* and *K. obovata* was consistent (**Figure 7D**), the marked differences in protein conformation fluctuated around the residues 70~80, 160~180, and 450 (**Figure 7E**).

**Figure 8:**
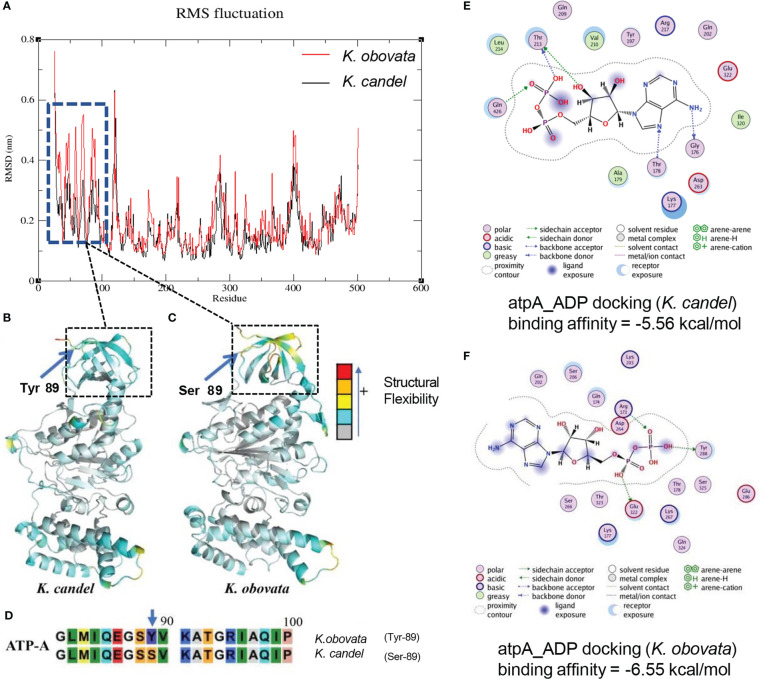
The predicated 3D structural modeling and molecular dimnamics simulation of the ATP-A protein. By setting different colors (gray to blue to yellow to orange to red) according to the b-factor values, the color in the structure is closer to red, the more flexible the structure. **(A)** The conformational variations of protein amino acid residues during the molecular dynamics simulation process showing the distinct difference in the region from 26aa to 96aa. **(B)** The 3D model of ATP-A protein for *K*. *candel*. **(C)** The 3D model of ATP-A protein for *K*. *obovata*. **(D)** The mutation at the site of the 89 aa of ATP-A protein between *K*. *candel* and *K*. *obovata*. **(E)** Molecular dimnamics simulation of ATP-A protein and ligand ADP docking exhibuted by binding affinity for *K*. *candel* **(E)** and *K. obovata* **(F)**.

The structure of ATP α subunit protein (ATP-A) coded by the gene *atpA* was constructed in the same way as the NDH-D protein (**Figure 8**). According to the SNP result, the Tyr89 in protein ATP-A in *K. candel* was mutated to the Ser89 in *K. obovata*, (**Figure 8D**). The predicted structures of ATP-A were shown in **Figures 8B, C**. RMSF analysis after MD simulations illustrated that great differences existed in the regions from the residue 26 to 96 amino-acid in ATP-A between species (**Figure 8A**). As the docking results showing in **Figures 8E, F**, a larger absolute value of binding affinity (-6.55 kcal/mol vs. -5.56 kcal/mol) indicated a stronger interaction between ATP-A and ADP in *K. obovata*, suggesting that the α subunit protein of ATP complex may work more actively in ATP synthesis in *K. obovata*.

*Kandelia* showed that *K. obovata* had a mutation site in ATP-A, while *K. candel* was identical at this site with others mangrove and Arabidopsis, which located in the protein domain (**Supplementary Figure S9**). Among these, *K. obovata* was consistent in the amino acid at the position of 22nd with *Arabidopsis* but different from *K. candel* and the other two Rhizophoraceae species, *Bruguiera sexangula* and *Rhizophora apiculata*. The sites of 22nd, 45th and 426th showed that *K. candel* and *A. thaliana* were consistent while *K.obovata* species were different from all the other compared species (**Supplementary Figure S9**).

The two animations for the whole 50ns MD simulations demonstrating the ATP-A-ADP binding are provided in the supplementary movie document (**Supplementary Animations A**, **B**).

## 4 Discussion

### 4.1 Comparative analysis of the 25 cp genomes gave support to *Kandelia* being differentiated into two distinct species

*Kandelia* (Rhizophoraceae) was regarded as a monotypic genus consisting of a single *K. candel* (Hou, 1958; Juncosa & Tomlinson, 1988). Based on studies on leaf anatomy, cold-resistance adaptation, chromosome number, and molecular markers from both cp and mitochondria, *K. obovata* was identified as a new mangrove species differing from *K. candel* 20 years ago (Sheue et al., 2003), but the genomic information between them remained unknown, which limited our understanding about how their genes evolved and differentiated during the process of plant speciation. This also causes confusion in scientific exchanges, as some scholars still use *K. candel* for the plant materials of *Kandelia* collected either from China or Japan (Geng et al., 2008; Enoki et al., 2009; Zhang et al., 2012a; Feng et al., 2022).

The plastid genome provides valuable information for species identification, population genetics, species differentiation, and phylogenetic analyses (Daniell et al., 2016; Kong et al., 2021; Luo et al., 2021). In this study, we obtained the high-quality cp genomes of *Kandelia* (**Table 1**, **Figure 3**), with the cp genome size ranging from 165,247 to 168,262 bp, which were almost 3 kb larger than previously published *K. obovata* cp genomes (Chen et al., 2019; Yang et al., 2019). The number of genes and arrangements were also different in comparison with previous studies. Genes *psbK* and *psbI* were assembled and annotated in our study, which were located in the aforementioned 3 kb regions, but they were absent in previous studies (Chen et al., 2019; Yang et al., 2019). To confirm the correctness of our cp genome assembly, we designed primers around gene *psbK* and double-checked the locations of *psbK* and *psbI* genes by randomly selecting seven samples with PCR amplification. The results verified that they were in the right sequence position in the cp genomes (**Supplementary Figure S1**). In addition, the fragment from 8,966 to 52,067 bp was in inverse order in the LSC region, which was different from the reported cp genome of *K. obovata* (Chen et al., 2019; Yang et al., 2019). This inversion has been previously reported in other cp genomes (Wei et al., 2020; Cui et al., 2021; Kim & Cheon, 2021; Wang et al., 2021).

The genomes of all samples shared 83 protein-coding genes, 37 transfer RNA genes, and eight ribosomal RNA genes. The size of the cp genome in *K. obovata*, however, was roughly 2 kb longer than that in *K. candel*, with the greatest difference in the LSC regions. The GC content in the IRs remained the same, but both LSC and SSC were slightly lower in *K. obovata* in comparison with *K. candel* (**Figure 3**, **Supplementary Table S4**).

Indels are an important class of genetic variations and play important roles in species evolution (Biju et al., 2019; Yang et al., 2019). We identified 1,522 indels in 25 cp genomes of *Kandelia*, comprising 112 SSR-related indels and 1,410 non-SSR-related indels (**Figure 4**). The phylogenetic tree based on the indel data of all cp genomes divided 25 samples into two separate branches. PCA analysis was in accordance with the phylogenetic tree established by the SNPs. Phylogenetic relationship analyses based on 76 homologous protein genes from the single-copy genes in these 25 cp genomes (**Figure 2A**) and 38 homologous pair genes among 13 mangrove species, including 40 samples (**Figure 2B**), provided strong support to the genus *Kandelia* consisting of two distinct species. These two phylogenetic analyses also coincided with the results obtained by the SNPs of the whole cp genome tree. Our integrative phylogenetic trees provided detailed molecular information revealing that *K. candel* and *K. obovata* are two species geographically separated by the South China Sea, as previously suggested by Sheue et al. (2003). In terms of *K. obovata* clustering into two subgroups, among the many possible causes, introduction or transplant was likely the main reason for gene homogenization. Among these, YQ in Zhejiang Province and LD in Hainan Province were the two artificial introduction places for *K. obovata*, and Fujian was the main provenance of the propagules of *K. obovata* (Li et al., 2001; Wang & Wang, 2007).

Repeat sequences are an important part of the genome and play an important role in the evolution of organisms, genetics, and regulation of gene expression. Comparison of the tandem, forward, and palindromic repeats markedly differed between *K. candel* and *K. obovata* (**Supplementary Figure S3**). Tandem and forward repeats in *K. obovata* were more frequent than those in *K. candel* (**Supplementary Figure S3B, C**). In contrast, palindromic repeats were less frequent in *K. obovata* than in *K. candel* (**Supplementary Figure S3D**). This also resulted from the differences in cp genome sizes between *K. candel* and *K. obovata*, due to these different repetitive sequences mainly located in the intergenic regions. Microsatellites, also known as simple sequence repeats (SSRs), are short repeating DNA sequences of one to six base pairs that are ubiquitous in the genome (Powell et al., 1996; Fang et al., 2020). Owing to the number of repeat units that may vary between individual genotypes, SSR is extremely versatile and useful for genetic analysis (Zalapa et al., 2012). In this study, more SSRs were found in *K. candel* than in *K. obovata*, especially the type of mononucleotide in the Malaysian samples (**Supplementary Figure S4**). Moreover, SSRs typically consist of a higher number of AT bases than GC bases (Wang et al., 2022), which is consistent with our observations and the high AT content in the nucleotide composition of *K. candel*. Given that SSRs were applied to create maps of genetic linkage, variety identification, and molecular markers (Gupta et al., 2022; Steele et al., 2006), detailed information on the SSRs identified in the present study can facilitate future research on selected target regions for more in-depth population studies between these two species within genus *Kandelia*.

### 4.2 Variations and non-synonymous mutations of the cp genomes between *K. candel* and *K. obovata*

Mauve analysis showed an indel with a length of 1,149 bp in the LSC region between *K. obovata* and *K. candel* (**Figure 5**), which explains the striking difference in cp genome sequence composition between *K. candel* and *K. obovata*. Near this region with a high nucleotide diversity value (within 2 kb upstream and downstream), there were non-synonymous mutations in the *atpA* and *ndhD* genes between *K. candel* and *K. obovata* (**Figure 6**). *MatK* and *rps4* had non-synonymous mutation sites in different samples of *K. candel*, while the *atpB* and *rbcL* genes had non-synonymous mutation sites in different samples of *K. obovata* (**Figure 6**). Moreover, the genes with a *Pi* value of >0.01 mentioned above were located in the LSC and SSC regions. Taking cp DNA *atpB*-*rbcL* spacer and *trnL*-*trnF* spacer as examples, both were used as universal cp DNA markers to identify the two geographically distributed populations of *Kandelia* (Chiang et al., 2001). In the present study, detailed information obtained by comparison of the 25 cp genomes of *Kandelia* indicated that there were 58 variant loci in *atpB*-*rbcL* and four variant loci in *trnL*-*trnF* spacers between the two species. As the cp genome has a copy-dependent repair mechanism, which in turn ensures the conservation and stability of the two IR regions in the cp genome (Khakhlova & Bock, 2006; Wang et al., 2022), the variation in the IR region was much less than in the LSC and SSC regions. Sequence variations existed throughout the cp genomes between the *K. candel* and *K. obovata*, especially the non-synonymous mutation sites between the two species. Predicting the mutation sites in non-synonymous amino acid residues would doubtlessly provide a foundation for further research on this protein in adaptation to habitat.

### 4.3 Structural and functional simulation analyses of non-synonymous proteins between *K. candel* and *K. obovata*

Light intensity and temperature are the two key environmental factors for differentiating *K. candel* and *K. obovata* geographically distributed in the northern and southern banks of the South China Sea, respectively. Adaptations to these key factors are the most critical characteristics of their differential genes and corresponding protein evolution in the northward colonization of *Kandelia*. Comparative analyses of sequence divergence and mutation hotspots revealed that the *atpA* gene in the LSC region and the *ndhD* gene in the SSC region exhibited distinct patterns between *K. candel* and *K. obovata* (**Figures 3**, **5**, **6**, **7C**, **8D**).

The cp NDH complex is composed of 11 plastid genes (*ndh* A~J) together with at least 18 nuclear genes that function as thylakoid NAD(P)H dehydrogenase (Shen et al., 2021). The complex is involved in electron transfer from NAD(P)H to plastoquinone, protecting the plant cell against photooxidative stress and maintaining optimal rates of photosystem I (PSI) cyclic photophosphorylation (Braukmann et al., 2009; Peredo et al., 2013). NDH-dependent cyclic electron flow (CEF) provides extra ΔpH and ATP for the CO_2_ assimilation, which is essential for balancing the changing demands for ATP/NADPH and regulating photosynthetic machinery in response to various environmental conditions (Zhu et al., 2001; Yukawa et al., 2005; Shikanai, 2016; Shikanai, 2020; Shen et al., 2021). Therefore, it functions as a value-feeding electron to poise the redox level of the intermediaries to optimize the rate of the cyclic electron transport in accordance with the changes in light intensity (Casano et al., 2000; Joet et al., 2002). More research has shown that *ndh* genes are of great importance in photosynthesis regulation in adaptation to harsh environments. Small changes in any of the *ndh* genes significantly decrease the photosynthesis rate (Endo et al., 1999; Silva et al., 2016). Adaptation to submersed environments is accompanied by the complete loss of the NDH complex in an aquatic angiosperm (Peredo et al., 2013). A series of T-to-C inactivating mutations occurred in *ndh* genes, which were further corrected back to T during evolution (Martín & Sabater, 2010), implying the importance of the *ndh* genes in the stability of the complex. Given the location of NDH-D in the transmembrane helix of the NDH complex, it plays a key role in maintaining the activity and plasticity of the complex (Li et al., 2013; Shen et al., 2021). Any variations in NDH-D would impact photosynthetic electron transport, energy generation, and light adaptation.

In the present study, the *ndh-D* gene showed three non-synonymous mutation sites, two of which mutated from base C in *K. candel* to base A in *K. obovata* and one from base G in *K. candel* to base A in *K. obovata*, which therefore caused the amino acids at three sites to vary from F (Phe), L (Leu), and M (Met) to L (Leu), I (Ile), and I (Ile) in *K. obovata*, showing intraspecific consistency but the obvious interspecific difference at the 22nd, 45th, and 426th amino acids in the protein sequence between the two species (**Figure 7**). Further comparative analysis of the structural modeling of the NDH-D protein between the two species illustrated that b-factors for the three sites were higher in *K. candel*, illustrating that the protein possessed higher flexibility at the three amino acid regions in comparison with *K. obovata* (**Figure 7**). As protein structural plasticity is closely related to its active function in light harvesting and photosynthetic electron transport (Tian et al., 2012; Sun et al., 2019), considering *K. candel* mainly distributed in Southeastern Asia with higher light intensity and temperature in comparison with *K. obovata* growing in Eastern Asia, we suggested this might relate to the long-term adaptation to differential light radiation in their respective habitats.

Temperature has a strong impact on protein evolution. It has been found that orthologous proteins of species evolved at different temperatures commonly exhibit distinctive differences in function and structural stability in adaptation to temperature (Fields et al., 2015; Cvetkovska et al., 2018). In chloroplasts, F_0_F_1_-ATP synthase (cp H^+^-ATPase) is a protein complex responsible for ATP synthesis and energy generation. It contains two components with independent functions, a membrane-intrinsic CF_0_, which is responsible for the proton flux across the membrane, and a membrane-extrinsic CF_1_, which catalyzes the synthesis of ATP from ADP and phosphate using the energy of electrochemical transmembrane potential of protons (Nelson & Ben-Shem, 2004; Hahn et al., 2018). The core component of CF_1_ is α3β3γ, consisting of three ATP-A (α) and three ATP-B (β) subunits, which are encoded by the chloroplast DNA (Rodermel & Bogorad, 1987). It has been reported that mutation or editing deficiency at a certain site results in a substitution of amino acid residue, impairing the assembly of chloroplast ATP synthase (Liu et al., 2021). A large number of studies have suggested that when exposed to lower temperatures, resistant plants showed higher ATPase activity than susceptible ones, which provide extra energy in plant responses to low temperatures (Gilmore & Bjrkman, 1995; Schttler & Toth, 2014). Ultracytochemical localization studies on the effect of ATPase on low-temperature adaptability also provide direct evidence that chloroplast ATPase is closely related to the plant’s tolerance to low temperatures (He, 2020). Our analysis showed that *K. obovata* had serine (S) at the 89th site in the ATP-A protein domain instead of tyrosine (Y), as in *K. candel* (**Figure 8D**). Molecular dynamics simulation indicated that the ATP-A protein of *K. obovata* had a higher binding ability with ADP (**Figures 8E, F**), which implied that its ATPase possessed higher efficiency in ATP formation. In addition, the gene *atpB* remained consistent within *K. candel* individuals but exhibited variations in SNP loci within the different populations of *K. obovata* (**Figure 6**). Hence, the mutation sites in the genes *atpA* and *atpB* of *K. obovata* populations might closely relate to their adaptation to the lower temperature in the northern part of the South China Sea, which endowed it with higher tolerance to lower temperature in the northward colonization of *K. obovata*.

The above results provide molecular proof for the divergence of *K. obovata* and *K. candel* in adaptation to different habitats. This also well explained the previous physiological experimental results obtained both from a common garden trial in Hong Kong (Maxwell, 1995) and in a lab chilling experiment conducted between these two *Kandelia* species (Short et al., 2021). In 2008, low air temperature in winter caused serious damage to several mangrove species, but *K. obovata* exhibited the highest cold tolerance among mangroves during extreme cold events in China (Chen et al., 2010), which could be more ecophysiological proof to support our conclusions gained from the cp genomes.

## 5 Conclusions

The main finding reported here first provides the whole cp genome of *K. candel*, and comparative analyses of the whole cp genomes were conducted between *K. candel* and *K. obovata*. Although the cp genome is regarded as having a highly conservative nature and a slow evolutionary rate, interspecific differences exist between *K. candel* and *K. obovata* in adaptation to differential environments geographically separated by the South China Sea, including the sizes of cp genomes, codon usages, repeat sequences, and indels of genes. Significant interspecific differences were found in *ndhD* and *atpA*, which are involved in photosynthetic electron transport and ATP formation, implying that energy generation plays a pivotal role in their ability to adapt to different temperatures and light, two main geographical environmental factors. Future comparative studies on photosynthetic electron transport and photophosphorylation caused by the genetic variations of *atpA* and *ndhD* in these species are needed to clarify their response mechanisms to varied light radiation and temperatures.

## Data availability statement

The datasets presented in this study can be found in online repositories. The names of the repository/repositories and accession number(s) can be found below: GenBank database under accession numbers from ON969308 to ON969332.

## Author contributions

XZ designed the research project. XX, YZ performed research. XX, QL, YS and XZ analyzed data. XX, XZ and YS wrote the manuscript. LC, WW, GC, WN, MI, PP, HZ prepared plant materials and contributed to scientific discussion and revision of the manuscript. All authors have read and approved the final manuscript.

## References

[B1] Alexander D. H. Novembre J. Lange K. (2009). Fast model-based estimation of ancestry in unrelated individuals. Genome Res. 19 (9), 1655–1664. doi: 10.1101/gr.094052.109 19648217 PMC2752134

[B2] Beier S. Thiel T. Münch T. Scholz U. Mascher M. (2017). MISA-web: A web server for microsatellite prediction. Bioinf. (Oxford England) 33 (16), 2583–2585. doi: 10.1093/bioinformatics/btx198 PMC5870701 28398459

[B3] Benson G. (1999). Tandem repeats finder: A program to analyze DNA sequences. Nucleic Acids Res. 27 (2), 573–580. doi: 10.1093/nar/27.2.573 9862982 PMC148217

[B4] Biju V. C. Vijayan S. Rajan V. S. Sasi A. Janardhanan A. Nair A. S. (2019). The complete chloroplast genome of trichopus zeylanicus, and phylogenetic analysis with dioscoreales. Plant Genome 12 (3), 1–11. doi: 10.3835/plantgenome2019.04.0032 PMC12810117 33016590

[B5] Birky C. W. (2001). The inheritance of genes in mitochondria and chloroplasts: Laws, mechanisms, and models. Annu. Rev. Genet. 35, 125–148. doi: 10.1146/annurev.genet.35.102401.090231 11700280

[B6] Bobik K. Burch-Smith T. M. (2015). Chloroplast signaling within, between and beyond cells. Front. Plant Sci. 6 (781). doi: 10.3389/fpls.2015.00781 PMC4593955 26500659

[B7] Bolger A. M. Lohse M. Usadel B. (2014). Trimmomatic: A flexible trimmer for illumina sequence data. Bioinf. (Oxford England) 30 (15), 2114–2120. doi: 10.1093/bioinformatics/btu170 PMC4103590 24695404

[B8] Braukmann T. W. A. Kuzmina M. Stefanovi S. (2009). Loss of all plastid ndh genes in gnetales and conifers: Extent and evolutionary significance for the seed plant phylogeny. Curr. Genet. 55 (3), 323–337. doi: 10.1007/s00294-009-0249-7 19449185

[B9] Burley S. K. Bhikadiya C. Bi C. Bittrich S. Chen L. Crichlow G. V.. (2021). RCSB protein data bank: powerful new tools for exploring 3D structures of biological macromolecules for basic and applied research and education in fundamental biology, biomedicine, biotechnology, bioengineering and energy sciences. Nucleic Acids Res. 49 (D1), D437–D451. doi: 10.1093/nar/gkaa1038 33211854 PMC7779003

[B10] Casano L. M. Zapata J. M. Martín M. Sabater B. (2000). Chlororespiration and poising of cyclic electron transport. Plastoquinone as electron transporter between thylakoid NADH dehydrogenase and peroxidase. J. Biol. Chem. 275 (2), 942–948. doi: 10.1074/jbc.275.2.942 10625631

[B11] Chen F. Mackey A. J. Stoeckert C. J. Roos D. S. (2006). OrthoMCL-DB: Querying a comprehensive multi-species collection of ortholog groups. Nucleic Acids Res. 34 (1), D363–D368. doi: 10.1093/nar/gkj123 16381887 PMC1347485

[B12] Chen C. Rui X. Hao C. He Y. (2018). Tbtools, a toolkit for biologists integrating various hts-data handling tools with a user-friendly interface. Cold Spring Harbor Lab. 8. doi: 10.1101/289660

[B13] Chen L. Z. Wang W. Q. Zhang Y. H. Huang L. Lin G. H. (2010). Damage to mangroves from extreme cold in early 2008 in southern China. Chin. J. Plant Ecol. 34 (2), 186–194. doi: 10.1016/S0140-6736(00)30105-2

[B14] Chen D. Q. Xiang S. Liu Z. J. Zou S. Q. (2019). The complete chloroplast genome sequence of kandelia obovata (Rhizophoraceae). Mitochondrial DNA Part B 4, 2, 3494–3495. doi: 10.1080/23802359.2019.1674745 33366055 PMC7707281

[B15] Chiang T. Y. Chiang Y. C. Chen Y. J. Chou C. H. Havanond S. Hong T. N.. (2001). Phylogeography of kandelia candel in East Asiatic mangroves based on nucleotide variation of chloroplast and mitochondrial DNAs. Mol. Ecol. 10 (11), 2697–2710. doi: 10.1046/j.0962-1083.2001.01399.x 11883883

[B16] Cui G. X. Wang C. M. Wei X. X. Wang H. B. Wang X. L. Zhu X. Q.. (2021). Complete chloroplast genome of hordeum brevisubulatum: Genome organization, synonymous codon usage, phylogenetic relationships, and comparative structure analysis. PloS One 16 (12), e0261196. doi: 10.1371/journal.pone.0261196 34898618 PMC8668134

[B17] Cvetkovska M. Szyszka-Mroz B. Possmayer M. Pittock P. Lajoie G. Smith D. R.. (2018). Characterization of photosynthetic ferredoxin from the Antarctic alga chlamydomonas sp. UWO241 reveals novel features of cold adaptation. New Phytol. 219 (2), 836–875. doi: 10.1111/nph.15194 29736931

[B18] Danecek P. Auton A. Abecasis G. Albers C. A. Banks E. DePristo M. A.. (2011). The variant call format and VCFtools. Bioinf. (Oxford England) 27 (15), 2156–2158. doi: 10.1093/bioinformatics/btr330 PMC3137218 21653522

[B19] Daniell H. Lin C. S. Yu M. Chang W. J. (2016). Chloroplast genomes: Diversity, evolution, and applications in genetic engineering. Genome Biol. 17 (1), 134. doi: 10.1186/s13059-016-1004-2 27339192 PMC4918201

[B20] Darling A. C. E. Mau B. Blattner F. R. Perna N. T. (2004). Mauve: Multiple alignment of conserved genomic sequence with rearrangements. Genome Res. 14 (7), 1394–1403. doi: 10.1101/gr.2289704 15231754 PMC442156

[B21] Das A. B. Basak U. C. Das P. (1995). Karyotype diversity and genomic variability in some Indian tree mangroves. Caryologia 48 (3–4), 319–328. doi: 10.1080/00087114.1995.10797341

[B22] Dierckxsens N. Mardulyn P. Smits G. (2020). Unraveling heteroplasmy patterns with NOVOPlasty. NAR Genomics Bioinf. 2 (1), lqz011. doi: 10.1093/nargab/lqz011 PMC7671380 33575563

[B23] Doyle J. (1991). DNA Protocols for plants: CTAB total DNA isolation. In Molecular techniques in taxonomy. (Springer : Berlin Heidelberg). 283–293.

[B24] Edgar R. C. (2004). MUSCLE: Multiple sequence alignment with high accuracy and high throughput. Nucleic Acids Res. 32 (5), 1792–1797. doi: 10.1093/nar/gkh340 15034147 PMC390337

[B25] Endo T. Shikanai T. Takabayashi A. Asada K. Sato F. (1999). The role of chloroplastic NAD(P)H dehydrogenase in photoprotection. FEBS Lett. 457 (1), 5–8. doi: 10.1016/S0014-5793(99)00989-8 10486552

[B26] Enoki T. Ueda M. Nanki D. Suwa R. Hagihara A. (2009). Distribution and stem growth patterns of mangrove species along the nakara river in iriomote island, southwestern Japan. J. For. Res. 14 (1), 51–54. doi: 10.1007/s10310-008-0094-4

[B27] Fang J. P. Lin A. T. Yuan X. Chen Y. Q. He W. J. Huang Y. J.. (2020). The complete chloroplast genome of isochrysis galbana and comparison with related haptophyte species. Algal Res. 50, 101989. doi: 10.1016/j.algal.2020.101989

[B28] Feng C. You H. M. Tan F. L. Han J. L. Yu X. X. You W. B.. (2022). Methane contributions of different components of kandelia candel–soil system under nitrogen supplementation. Forests 13 (2), 318. doi: 10.3390/f13020318

[B29] Fields P. A. Dong Y. Meng X. Somero G. N. (2015). Adaptations of protein structure and function to temperature: there is more than one way to 'skin a cat'. J. Exp. Biol. 218 (Pt 12), 1801–1811. doi: 10.1242/jeb.114298 26085658

[B30] Geng Q. Lian C. Goto S. Tao J. Kimura M. Islam M. S.. (2008). Mating system, pollen and propagule dispersal, and spatial genetic structure in a high-density population of the mangrove tree kandelia candel. Mol. Ecol. 17 (21), 4724–4739. doi: 10.1111/j.1365-294X.2008.03948.x 19140988

[B31] Giang L. H. Geada G. L. Hong P. N. Tuan M. S. Lien N. T. H. Ikeda S.. (2006). Genetic variation of two mangrove species in kandelia (Rhizophoraceae) in Vietnam and surrounding area revealed by microsatellite markers. Int. J. Plant Sci. 167 (2), 291–298. doi: 10.1086/499611

[B32] Gilmore A. M. Bjrkman O. (1995). Temperature-sensitive coupling and uncoupling of ATPase-mediated, nonradiative energy dissipation: Similarities between chloroplasts and leaves. Planta 197 (4), 646–654. doi: 10.1007/BF00191573

[B33] Greiner S. Lehwark P. Bock R. (2019). OrganellarGenomeDRAW (OGDRAW) version 1.3.1: Expanded toolkit for the graphical visualization of organellar genomes. Nucleic Acids Res. 47 (W1), W59–W64. doi: 10.1093/nar/gkz238 30949694 PMC6602502

[B34] Groth G. Pohl E. (2001). The structure of the chloroplast F1-ATPase at 3.2 a resolution. J. Biol. Chem. 276, 1345–1352. doi: 10.1074/jbc.M008015200 11032839

[B35] Gupta P. K. Balyan H. S. Edwards K. J. Isaac P. Korzun V. Rder M.. (2022). Genetic mapping of 66 new microsatellite (SSR) loci in bread wheat. TAG. theoretical and applied genetics. Theor. Appl. Genet. 105 (2–3), 413–422. doi: 10.1007/s00122-002-0865-9 12582546

[B36] Hahn A. Vonck J. Mills D. J. Meier T. Kühlbrandt W. (2018). Structure, mechanism, and regulation of the chloroplast ATP synthase. Science 360 (6389), eaat4318. doi: 10.1126/science.aat4318 29748256 PMC7116070

[B37] He J. Y. (2020). Study on the effect of ATPase on the low-temperature adaptability of galinsoga parviflora cav. by ultracytochemical localization. J. Electron Microscopy 39 (03), 307–312. doi: 10.3969/j.issn.1000-6281.2020.03.013

[B38] Hou D. (1958). “Flora malesiana,” in Rhizophoraceae Ed. van Steenis C. G. G. J.(Djakarta: Noordhoff-ko lffN.V.), pp 429–493.

[B39] Jin J. J. Yu W. B. Yang J. B. Song Y. dePamphilis C. W. Yi T. S.. (2020). GetOrganelle: A fast and versatile toolkit for accurate *de novo* assembly of organelle genomes. Genome Biol. 21 (1), 241. doi: 10.1186/s13059-020-02154-5 32912315 PMC7488116

[B40] Joet T. Cournac L. Peltier G. Havaux M. (2002). Cyclic electron flow around photosystem I in C(3) plants. *In vivo* control by the redox state of chloroplasts and involvement of the NADH-dehydrogenase complex. Plant Physiol. 128 (2), 760–769. doi: 10.1104/pp.010775 11842179 PMC148937

[B41] Johnson M. Mark J. Irena Z. Yan R. Yuri M. Scott M. G.. (2008). NCBI BLAST: a better web interface. Nucleic Acids Res. 36, W5–W9. doi: 10.1093/nar/gkn201 18440982 PMC2447716

[B42] Juncosa A. M. Tomlinson P. B. (1988). A historical and taxonomic synopsis of rhizophoraceae and anisophylleaceae. Ann. Missouri Botanical Garden 75), 1278–1295. doi: 10.2307/2399286

[B43] Katoh K. Standley D. M. (2013). MAFFT multiple sequence alignment software version 7: Improvements in performance and usability. Mol. Biol. Evol. 30 (4), 772–780. doi: 10.1093/molbev/mst010 23329690 PMC3603318

[B44] Khakhlova O. Bock R. (2006). Elimination of deleterious mutations in plastid genomes by gene conversion. Plant Journal: For Cell Mol. Biol. 46 (1), 85–94. doi: 10.1111/j.1365-313X.2006.02673.x 16553897

[B45] Kim K. A. Cheon K. S. (2021). Complete chloroplast genome sequence of adenophora racemosa (Campanulaceae): Comparative analysis with congeneric species. PloS One 16 (3), e0248788. doi: 10.1371/journal.pone.0248788 33735287 PMC7971521

[B46] Kong B. L. H. Park H. S. Lau T. W. D. Lin Z. Yang T. J. Shaw P. C. (2021). Comparative analysis and phylogenetic investigation of Hong Kong ilex chloroplast genomes. Sci. Rep. 11 (1), 5153. doi: 10.1038/s41598-021-84705-9 33664414 PMC7933167

[B47] Kumar S. Stecher G. Li M. Knyaz C. Tamura K. (2018). MEGA X: Molecular evolutionary genetics analysis across computing platforms. Mol. Biol. Evol. 35 (6), 1547–1549. doi: 10.1093/molbev/msy096 29722887 PMC5967553

[B48] Kurtz S. Choudhuri J. V. Ohlebusch E. Schleiermacher C. Stoye J. Giegerich R. (2001). REPuter: The manifold applications of repeat analysis on a genomic scale. Nucleic Acids Res. 29 (22), 4633–4642. doi: 10.1093/nar/29.22.4633 11713313 PMC92531

[B49] Langmead B. Salzberg S. L. (2012). Fast gapped-read alignment with bowtie 2. Nat. Methods 9 (4), 357–359. doi: 10.1038/nmeth.1923 22388286 PMC3322381

[B50] Letunic I. Khedkar S. Bork P. (2021). SMART: Recent updates, new developments and status in 2020. Nucleic Acids Res. 49 (D1), D458–D460. doi: 10.1093/nar/gkaa937 33104802 PMC7778883

[B51] Liao B. Zhang Q. (2014). Area, distribution and species composition of mangroves in China. Chin. J. Wetland Sci. 4 (12), 435–440. doi: 10.13248/j.cnki.wetlandsci.2014.04.005

[B52] Li H. Durbin R. (2009). Fast and accurate short read alignment with burrows-wheeler transform. Bioinformatics 25 (14), 1754–1760. doi: 10.1093/bioinformatics/btp324 19451168 PMC2705234

[B53] Li Q. H. He Z. H. Mi H. L. (2013). The research progress of chloroplast NAD(P)H dehydrogenase (NDH) complex. Chin. J. Plant Physiol. J. 49 (5), 401–409. doi: 10.13592/j.cnki.ppj.2013.05.008

[B54] Li M. S. Lee S. Y. (1997). Mangroves of China: A brief review. For. Ecol. Manage. 96 (3), 241–259. doi: 10.1016/S0378-1127(97)00054-6

[B55] Lin P. (1999). Mangrove ecosystem in China (Beijing: Science Press).

[B56] Liu X. Y. Jiang R. C. Wang Y. Tang J. J. Tan B. C. (2021). ZmPPR26, a DYW-type pentatricopeptide repeat protein, is required for c-to-U RNA editing at atpA-1148 in maize chloroplasts. J. Exp. Bot. 72 (13), 4809-4821. doi: 10.1093/jxb/erab185 33929512

[B57] Li J. Q. Xu H. F. Ye L. Z. Gu J. F. Wang R. Q. (2001). Introduction and afforestation technique of Kandelia candel to the North. Zhejiang Forestry Science and Technology 6, 51–53. Available at: https://www.docin.com/p-1526668739.html

[B58] Luo C. Huang W. L. Sun H. Y. Yer H. Y. Li X. Y. Li Y.. (2021). Comparative chloroplast genome analysis of impatiens species (Balsaminaceae) in the karst area of China: Insights into genome evolution and phylogenomic implications. BMC Genomics 22 (1), 571. doi: 10.1186/s12864-021-07807-8 34303345 PMC8310579

[B59] Martín M. Sabater B. (2010). Plastid ndh genes in plant evolution. Plant Physiol. Biochem. 48 (8), 636–645. doi: 10.1016/j.plaphy.2010.04.009 20493721

[B60] Maxwell G. S. (1995). Ecogeographic variation in from Brunei, Hong Kong and Thailand. Asia-Pacific Symposium Mangrove Ecosyst. 106, 59–65. doi: 10.1007/978-94-011-0289-6_8

[B61] McKenna A. Hanna M. Banks E. Sivachenko A. Cibulskis K. Kernytsky A.. (2010). The genome analysis toolkit: A MapReduce framework for analyzing next-generation DNA sequencing data. Genome Res. 20 (9), 1297–1303. doi: 10.1101/gr.107524.110 20644199 PMC2928508

[B62] Naskar K. R. Mandal R. N. (1999). Ecology and biodiversity of Indian mangroves. global status. (Delhi: Daya Publishing House 2, 397–400.

[B63] Nathan S. Andreas P. E. Alexandra C. Sereina R. Moritz W. Alan E. M.. (2011). Definition and testing of the GROMOS force-field versions 54A7 and 54B7. Eur. Biophysics J. 40, 843–856. doi: 10.1007/s00249-011-0700-9 21533652

[B64] Nelson N. Ben-Shem A. (2004). The complex architecture of oxygenic photosynthesis. Nat. Rev. Mol. Cell Biol. 5 (12), 971. doi: 10.1038/nrm1525 15573135

[B65] Peredo E. L. King U. M. Les D. H. (2013). The plastid genome of najas flexilis: Adaptation to submersed environments is accompanied by the complete loss of the NDH complex in an aquatic angiosperm. PloS One 8 (7), e68591. doi: 10.1371/journal.pone.0068591 23861923 PMC3701688

[B66] Powell W. Machray G. C. Provan J. (1996). Polymorphism revealed by simple sequence repeats. Trends Plant Sci. 1 (7), 215–222. doi: 10.1016/1360-1385(96)86898-1

[B67] Rice P. Longden I. Bleasby A. (2000). EMBOSS: The European molecular biology open software suite. Trends Genetics: TIG 16 (6), 276–277. doi: 10.1016/s0168-9525(00)02024-2 10827456

[B68] Rodermel S. R. Bogorad L. (1987). Molecular evolution and nucleotide sequences of the maize plastid genes for the alpha subunit of CF1 (atpA) and the proteolipid subunit of CF0 (atpH). Genetics 116 (1), 127–139. doi: 10.1093/genetics/116.1.127 2885245 PMC1203111

[B69] Rousseau Gueutin M. Bellot S. Martin G. E. Boutte J. Chelaifa H. Lima O.. (2015). The chloroplast genome of the hexaploid spartina maritima (Poaceae, chloridoideae): Comparative analyses and molecular dating. Mol. Phylogenet. Evol. 93, 5–16. doi: 10.1016/j.ympev.2015.06.013 26182838

[B70] Rozas J. Ferrer-Mata A. Sánchez-DelBarrio J. C. Guirao-Rico S. Librado P. Ramos-Onsins S. E.. (2017). DnaSP 6: DNA sequence polymorphism analysis of Large data sets. Mol. Biol. Evol. 34 (12), 3299–3302. doi: 10.1093/molbev/msx248 29029172

[B71] Sato S. Nakamura Y. Kaneko T. Asamizu E. Tabata S. (1999). Complete structure of the chloroplast genome of arabidopsis thaliana. DNA Research: Int. J. Rapid Publ. Rep. Genes Genomes 6 (5), 283–290. doi: 10.1093/dnares/6.5.283 10574454

[B72] Schttler M. A. Toth S. Z. (2014). Photosynthetic complex stoichiometry dynamics in higher plants: Environmental acclimation and photosynthetic flux control. Front. Plant Sci. 5 (188). doi: 10.3389/fpls.2014.00188 PMC4026699 24860580

[B73] Schuller J. M. Birrell J. A. Tanaka H. Konuma T. Wulfhorest H. Cox N.. (2019). Structural adaptations of photosynthetic complex I enable ferredoxin-dependent electron transfer. Science 363, 257–260. doi: 10.1126/science.aau3613 30573545

[B74] Shen L. L. Tang K. L. Wang W. D. Wang C. Wu H. Mao Z. Y.. (2021). Architecture of the chloroplast PSI-NDH supercomplex in hordeum vulgare. Nature 601, 649–654. doi: 10.1038/s41586-021-04277-6 34879391

[B75] Sheue C. R. Liu H. Y. Yong J. W. H. (2003). *Kandelia obovata* (Rhizophoraceae), a new mangrove species from Eastern Asia. Taxon 52, 2, 287–294. doi: 10.2307/3647398

[B76] Shi L. C. Chen H. M. Jiang M. Wang L. Q. Wu X. Huang L. F.. (2019). CPGAVAS2, an integrated plastome sequence annotator and analyzer. Nucleic Acids Res. 47 (W1), W65–W73. doi: 10.1093/nar/gkz345 31066451 PMC6602467

[B77] Shikanai T. (2016). Chloroplast NDH: A different enzyme with a structure similar to that of respiratory NADH dehydrogenase. Biochim. Biophys. Acta (BBA) - Bioenergetics 1857 (7), 1015–1022. doi: 10.1016/j.bbabio.2015.10.013 26519774

[B78] Shikanai T. (2020). Regulation of photosynthesis by cyclic electron transport around photosystem I. Adv. Botanical Res 96, 177–204. doi: 10.1016/bs.abr.2020.07.005

[B79] Short A. W. Chen R. Wee A. (2021). Comparison between parapatric mangrove sister species revealed higher photochemical efficiency in subtropical than tropical coastal vegetation under chilling stress. Aquat. Bot. 168, 103323. doi: 10.1016/j.aquabot.2020.103323

[B80] Silva S. R. Diaz Y. C. A. Penha H. A. Pinheiro D. G. Fernandes C. C. Miranda V. F. O.. (2016). The chloroplast genome of utricularia reniformis sheds light on the evolution of the ndh gene complex of terrestrial carnivorous plants from the lentibulariaceae family. PloS One 11 (10):e0165176. doi: 10.1371/journal.pone.0165176 27764252 PMC5072713

[B81] Steele K. A. Price A. H. Shashidhar H. E. Witcombe J. R. (2006). Marker-assisted selection to introgress rice QTLs controlling root traits into an Indian upland rice variety. TAG. theoretical and applied genetics. Theoretische Und Angewandte Genetik 112 (2), 208–221. doi: 10.1007/s00122-005-0110-4 16208503

[B82] Sun Z. T. Liu Q. Qu G. Feng Y. Reetz M. T. (2019). Utility of b-factors in protein science: Interpreting rigidity, flexibility, and internal motion and engineering thermostability. Chem. Rev. 119 (3), 1626–1665. doi: 10.1021/acs.chemrev.8b00290 30698416

[B83] Takeuchi T. Sugaya T. Kanazashi A. Katsuta Y. M. (2001). Genetic diversity of kandelia candel and bruguiera gymnorrhiza in the southwest islands, Japan. J. For. Res. 6 (3), 157–162. doi: 10.1007/BF02767087

[B84] Tian J. Wang P. Ning F. W. U. (2012). Recent advances in the rational design to improve the protein thermostability. Chin. J. Curr. Biotechnol. 2 (04), 233–239. doi: 10.3969/j.issn.2095-2341.2012.04.01

[B85] Tillich M. Lehwark P. Pellizzer T. Ulbricht-Jones E. S. Fischer A. Bock R.. (2017). GeSeq–versatile and accurate annotation of organelle genomes. Nucleic Acids Res. 45 (W1), W6–W11. doi: 10.1093/nar/gkx391 28486635 PMC5570176

[B86] Tomlinson P. B. (1986). The botany of mangroves (Cambridge University Press).

[B87] Van Der Spoel D. Lindahl E. Hess B. Groenhof G. Mark A. E. Berendsen H. J. C. (2005). GROMACS: Fast, flexible, and free. J. Comput. Chem. 26 (16), 1701–1718. doi: 10.1002/jcc.20291 16211538

[B88] Wang N. J. Chen S. F. Xie L. Wang L. Feng Y. Y. Lv T.. (2022). The complete chloroplast genomes of three hamamelidaceae species: Comparative and phylogenetic analyses. Ecol. Evol. 12 (2), e8637. doi: 10.1002/ece3.8637 35222983 PMC8848467

[B89] Wang C. X. Liu J. J. Su Y. Li M. L. Xie X. Y. Su J. J. (2021). Complete chloroplast genome sequence of sonchus brachyotus helps to elucidate evolutionary relationships with related species of asteraceae. BioMed. Res. Int. 2021, 9410496. doi: 10.1155/2021/9410496 34901281 PMC8654571

[B90] Wang W. Q. Wang M. (2007). The mangroves of China (Beijing: Scientific Press).

[B91] Wang D. P. Zhang Y. B. Zhang Z. Zhu J. Yu J. (2010). KaKs_Calculator 2.0: A toolkit incorporating gamma-series methods and sliding window strategies. Genomics Proteomics Bioinf. 8 (1), 77–80. doi: 10.1016/S1672-0229(10)60008-3 PMC5054116 20451164

[B92] Wei F. Tang D. F. Wei K. H. Qin F. Li L. X. Lin Y.. (2020). The complete chloroplast genome sequence of the medicinal plant sophora tonkinensis. Sci. Rep. 10 (1), 12473. doi: 10.1038/s41598-020-69549-z 32719421 PMC7385175

[B93] Yang L. C. Xiong F. Xiao Y. M. Li J. J. Chen C. Zhou G. Y.. (2019a). The complete chloroplast genome of swertia tetraptera and phylogenetic analysis. Mitochondrial DNA. Part B, Resources 5 (1), 164–165. doi: 10.1080/23802359.2019.1698368 33366469 PMC7748592

[B94] Yang Y. Zhang Y. Chen Y. K. Gul J. M. Zhang J. W. Liu Q.. (2019b). Complete chloroplast genome sequence of the mangrove species kandelia obovata and comparative analyses with related species. PeerJ 7, e7713. doi: 10.7717/peerj.7713 31579601 PMC6756139

[B95] Yoshioka H. Kondo K. Segawa M. Nehira K. Maeda S. (1984). Karyomorphological studies in five species of mangrove genera in the rhizophoraceae. Kromosomo 2 (35–36), 1111–1116.

[B96] Yukawa M. Tsudzuki T. Sugiura M. (2005). The 2005 version of the chloroplast DNA sequence from tobacco (Nicotiana tabacum). Plant Mol. Biol. Rep. 23 (4), 359–365. doi: 10.1007/BF02788884

[B97] Zalapa J. E. Cuevas H. Zhu H. Steffan S. Senalik D. Zeldin E.. (2012). Using next-generation sequencing approaches to isolate simple sequence repeat (SSR) loci in the plant sciences. Am. J. Bot. 99 (2), 193–208. doi: 10.3732/ajb.1100394 22186186

[B98] Zhang F. Q. Wang Y. S. Sun C. C. Lou Z. P. Dong J. D. (2012a). A novel metallothionein gene from a mangrove plant kandelia candel. Ecotoxicology 21 (6), 1633–1641. doi: 10.1007/s10646-012-0952-x 22711547

[B99] Zhang Z. Xiao J. F. Wu J. Y. Zhang H. Y. Liu G. M. Wang X. M.. (2012b). ParaAT: A parallel tool for constructing multiple protein-coding DNA alignments. Biochem. Biophys. Res. Commun. 419 (4), 779–781. doi: 10.1016/j.bbrc.2012.02.101 22390928

[B100] Zhu X. Y. Chen G. C. Zhang C. L. (2001). Photosynthetic electron transport, photophosphorylation, and antioxidants in two ecotypes of reed (Phragmites communis trin.) from different habitats. Photosynthetica 39 (2), 183–189. doi: 10.1023/A:1013766722604

