# Supplementary material for: A comparison of 25 complete chloroplast genomes between sister mangrove species Kandelia obovata and Kandelia candel geographically separated by the South China Sea
Source: Front Plant Sci. 2023 Jan 4;13:1075353. doi: 10.3389/fpls.2022.1075353 (PMC9845719; doi:10.3389/fpls.2022.1075353)
Supplement: Supplementary file 1 [file DataSheet_1.docx]

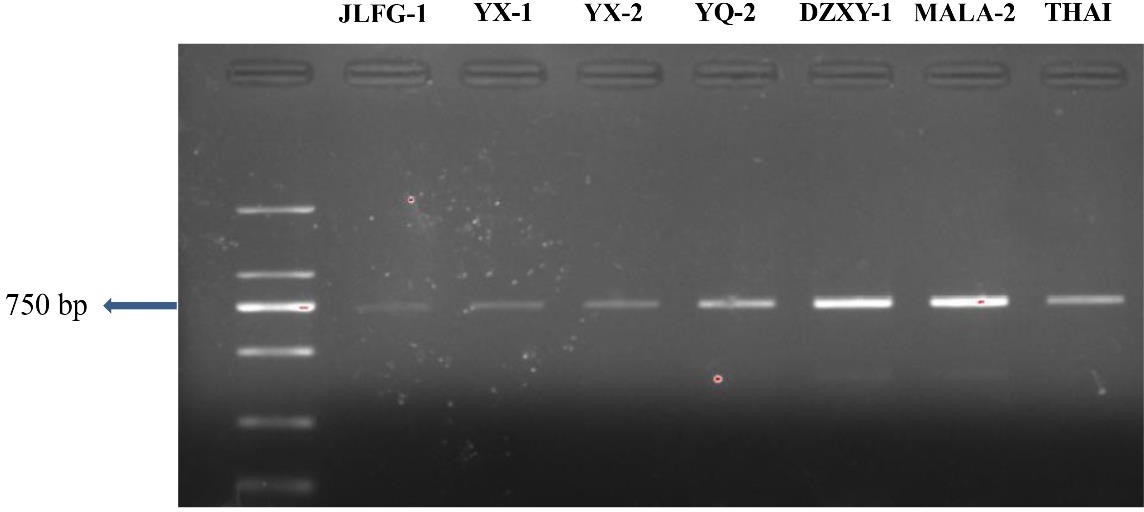


Figure S1. The PCR experiment was applied to check the assembly results. The randomly selected samples JLFG-1, YX-1, YX-2, YQ-2, DZXY-1, MALA-2 and THAI samples were used for confirmation with PCR.


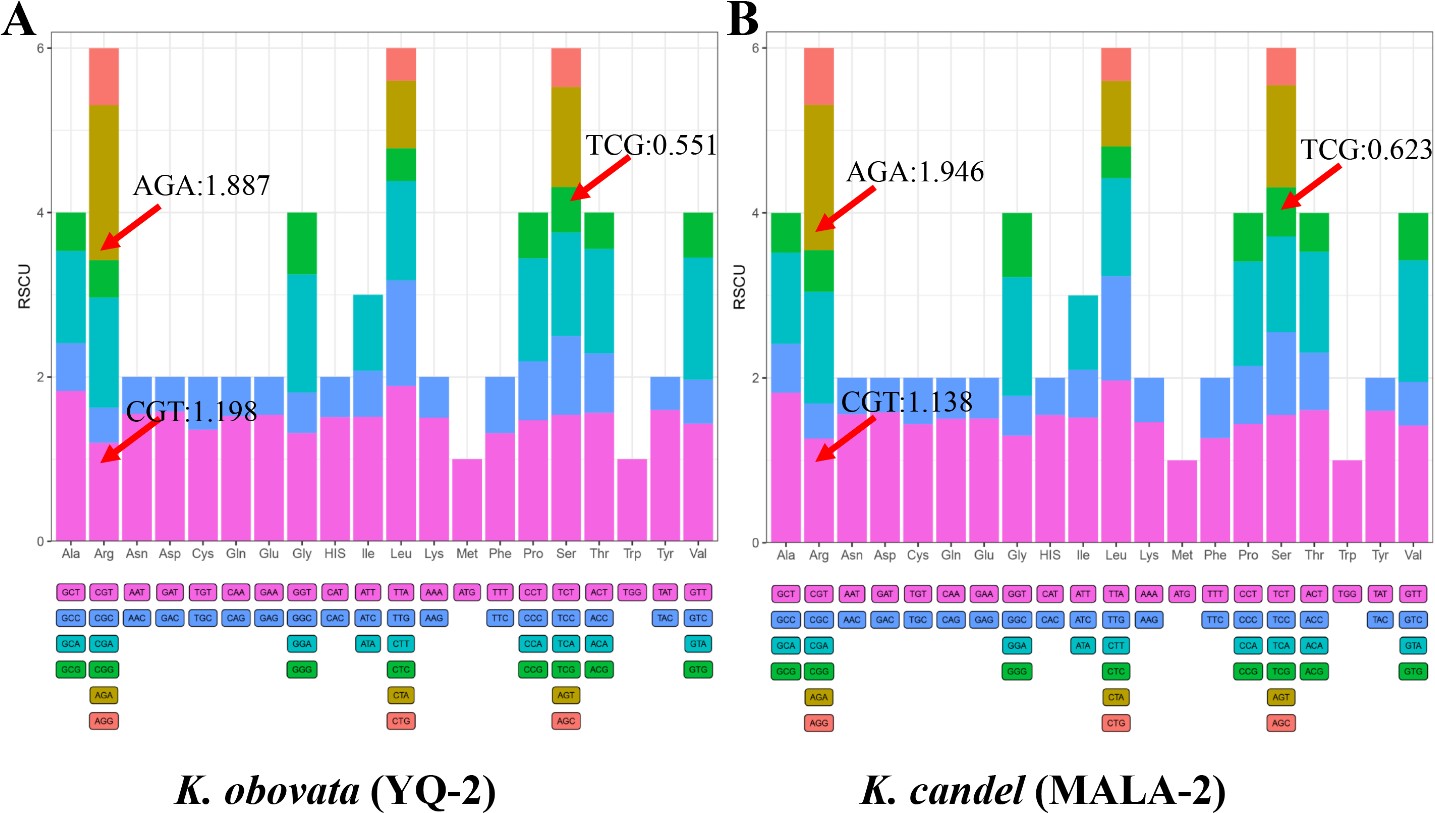


Fugure S2. Condon contents of 20 amino acids and stop condons in the protein-coding genes in the cp genomes of the *K. obovata* (A) and *K. candel* (B). Color of the histogram represent the proportions of codon usage for amino acid and stop codon. The arrow number means the relative synonymous codon usage number(RSCU).Arrows point to the top three differential condons of amino acids.


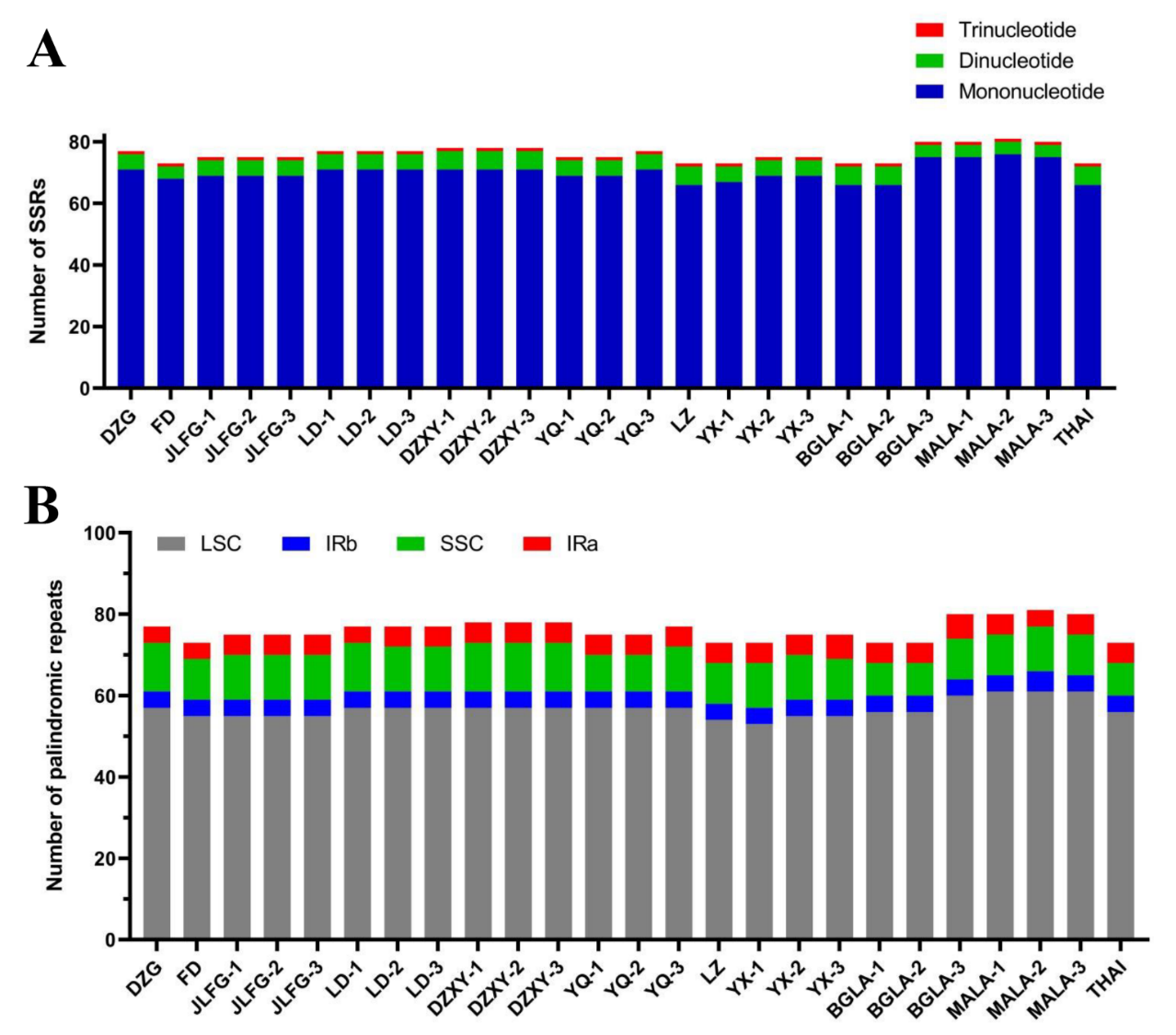


Figure S3. Simple sequence repeats (SSRs) analysis in the chloroplast genomes of *K. candel* and *K. obovata*. (A) Numbers of SSR types in complete chloroplast genomes. (B) Number of SSRs in LSC, IR and SSC regions.


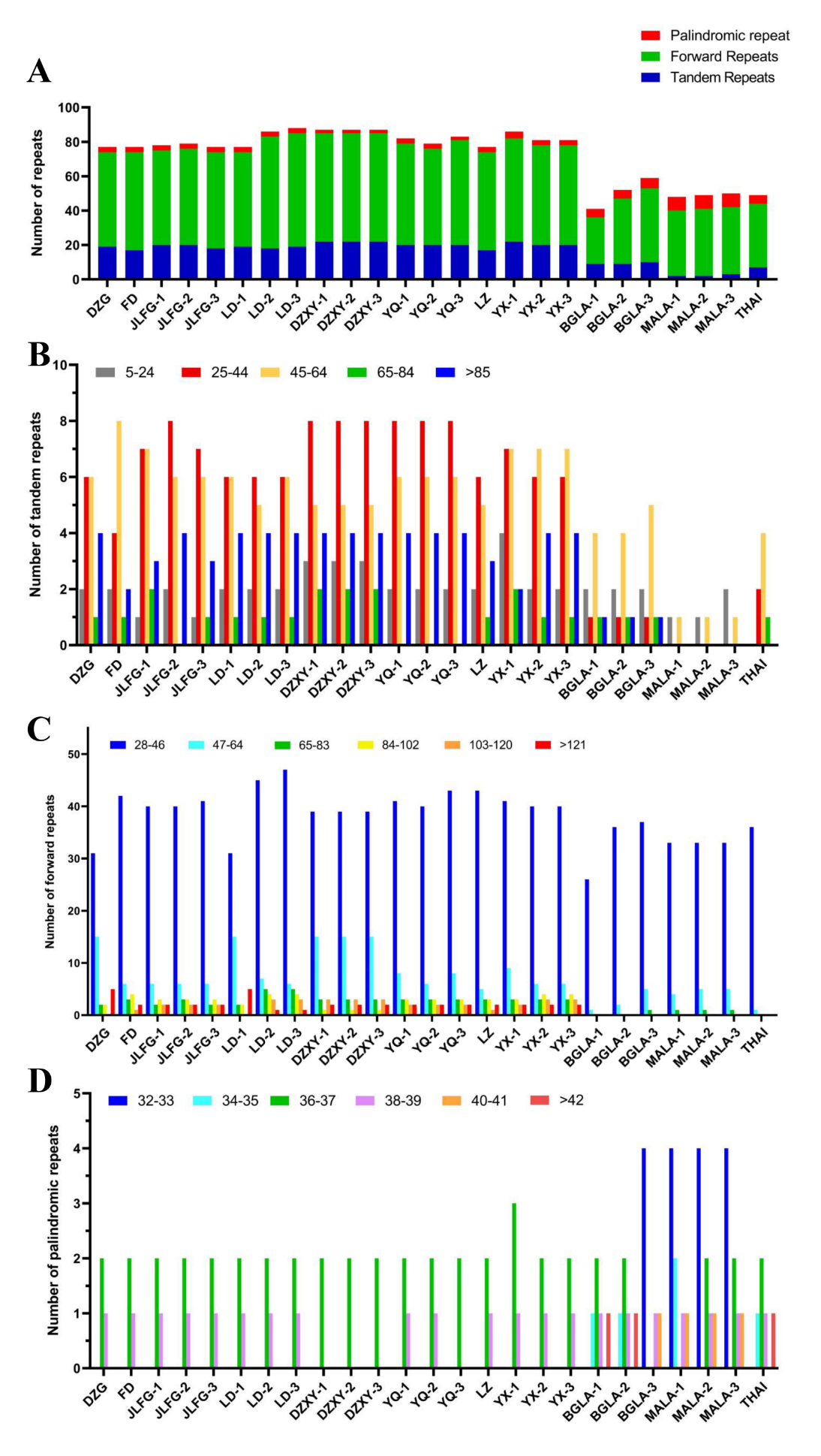


Figure S4. Distribution and frequency analysis of repeated sequences in chloroplast genome of *K. candel* and *K. obovata*. (A) Total number of palindromic, forward, and tandem repeats. (B) Distribution of tandem repeats in different ranges of length. (C) Distribution of forward repeats in different ranges of length. (D) Distribution of palindromic repeats in different ranges of length.


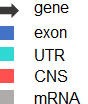


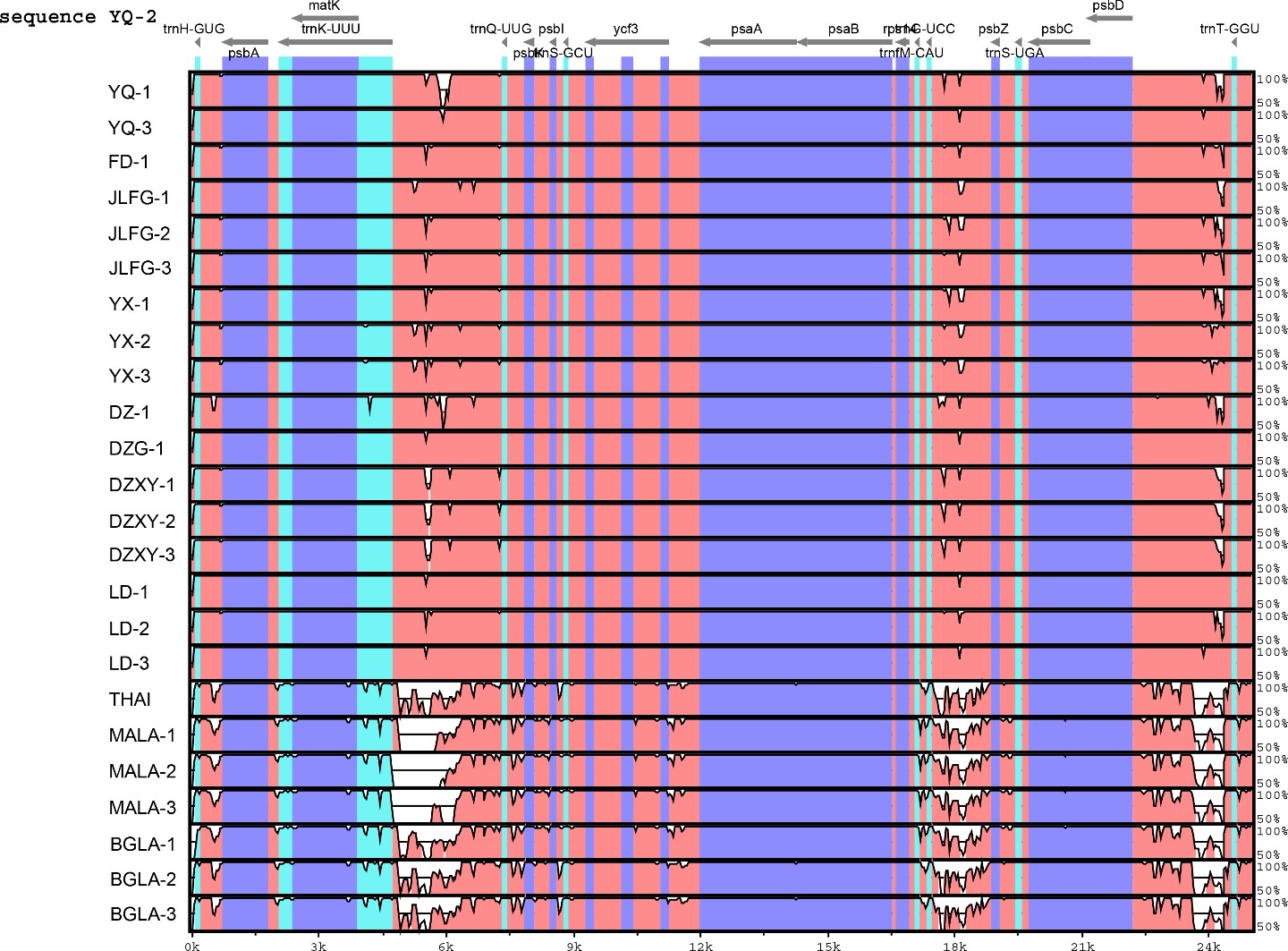


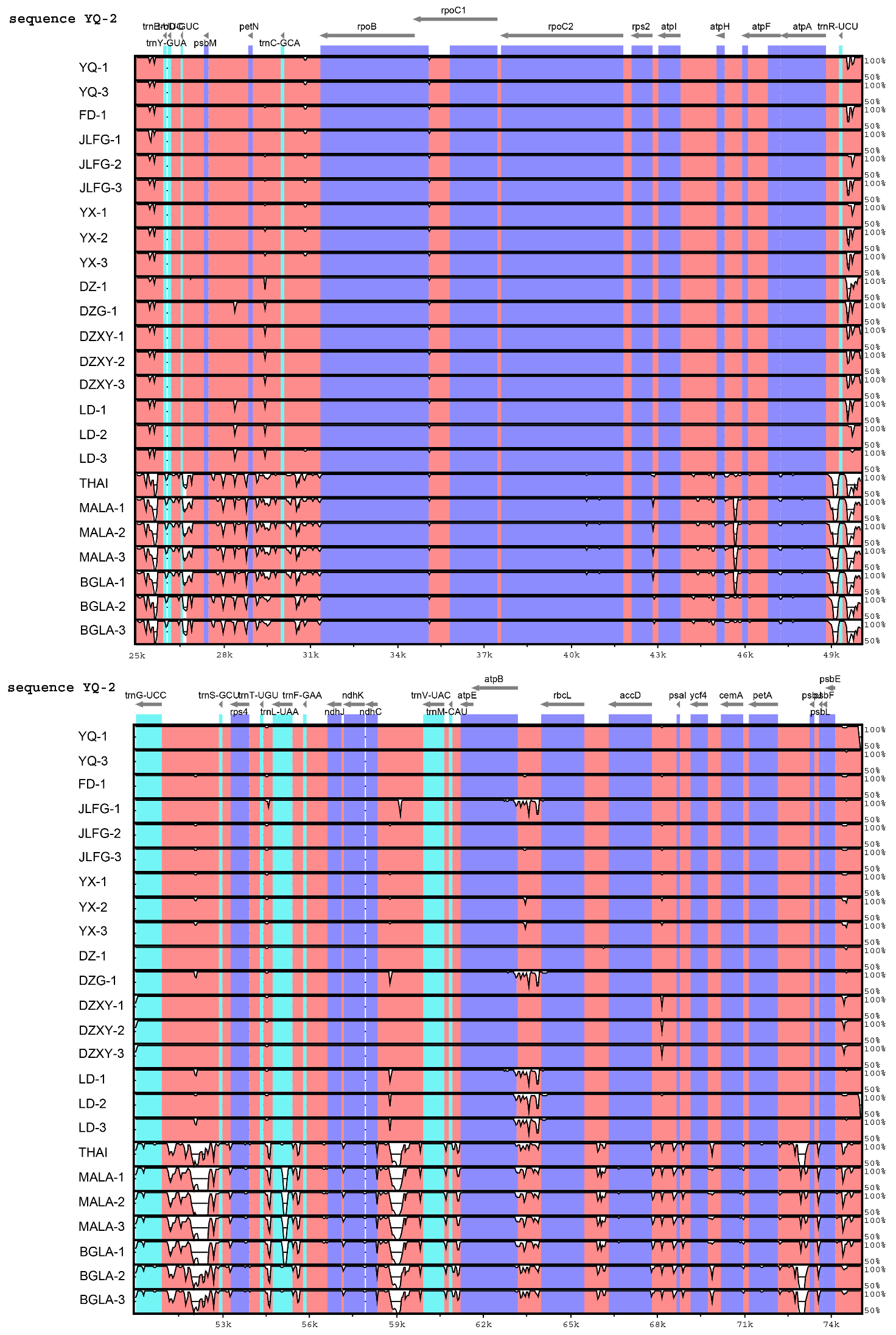

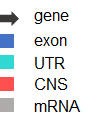

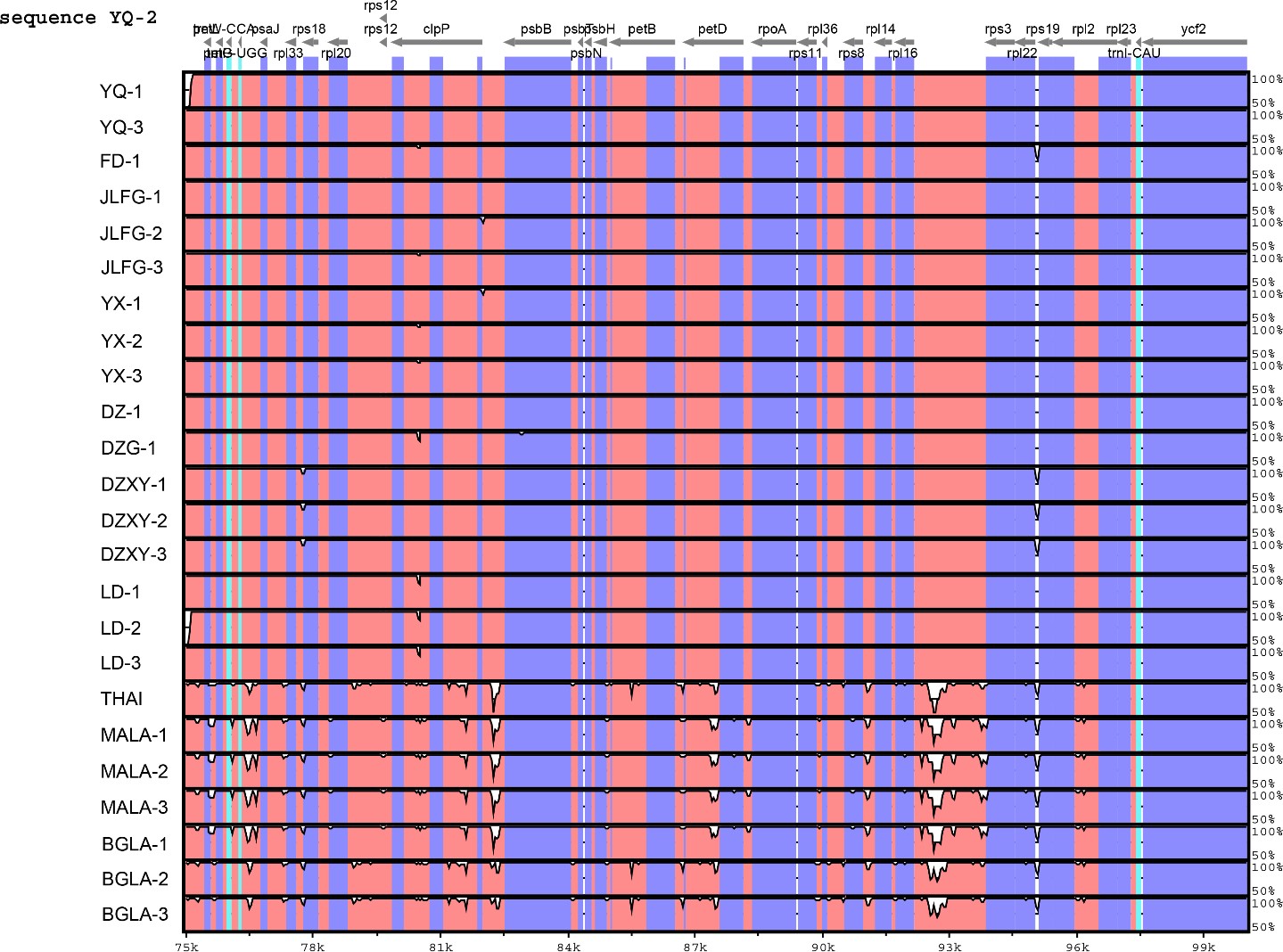

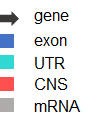

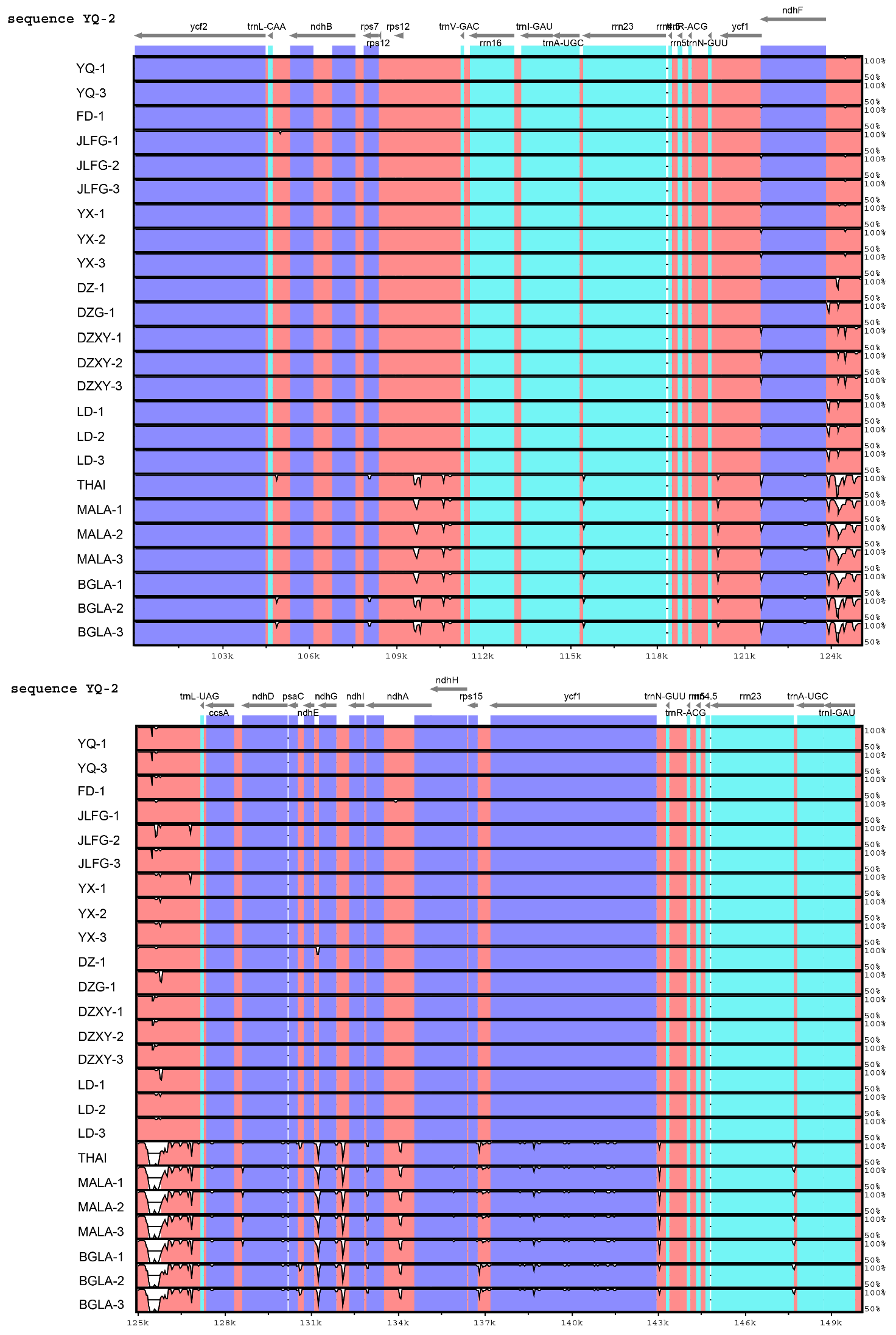

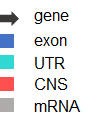


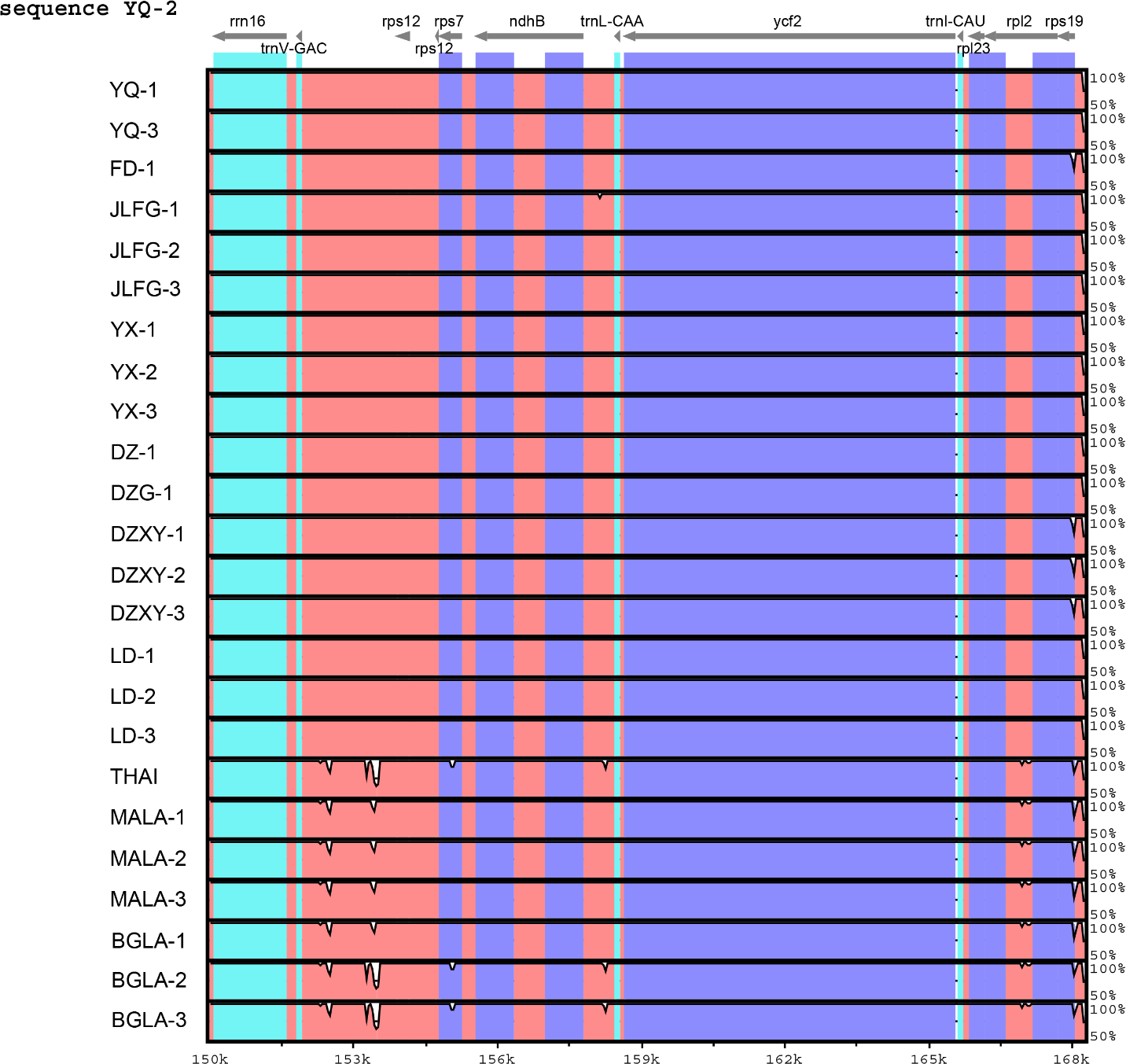

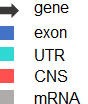


Figure S5. Complete chloroplast genome alignments of 25 samples using the mVISTA program, with the chloroplast genome of YQ-2 (*K. obovata*) as a reference. Arrows indicate the annotated genes and their transcriptional direction. Genome regions are color coded as exon, untranslated region (UTR), conserved non-coding sequences (CNS), and mRNA.


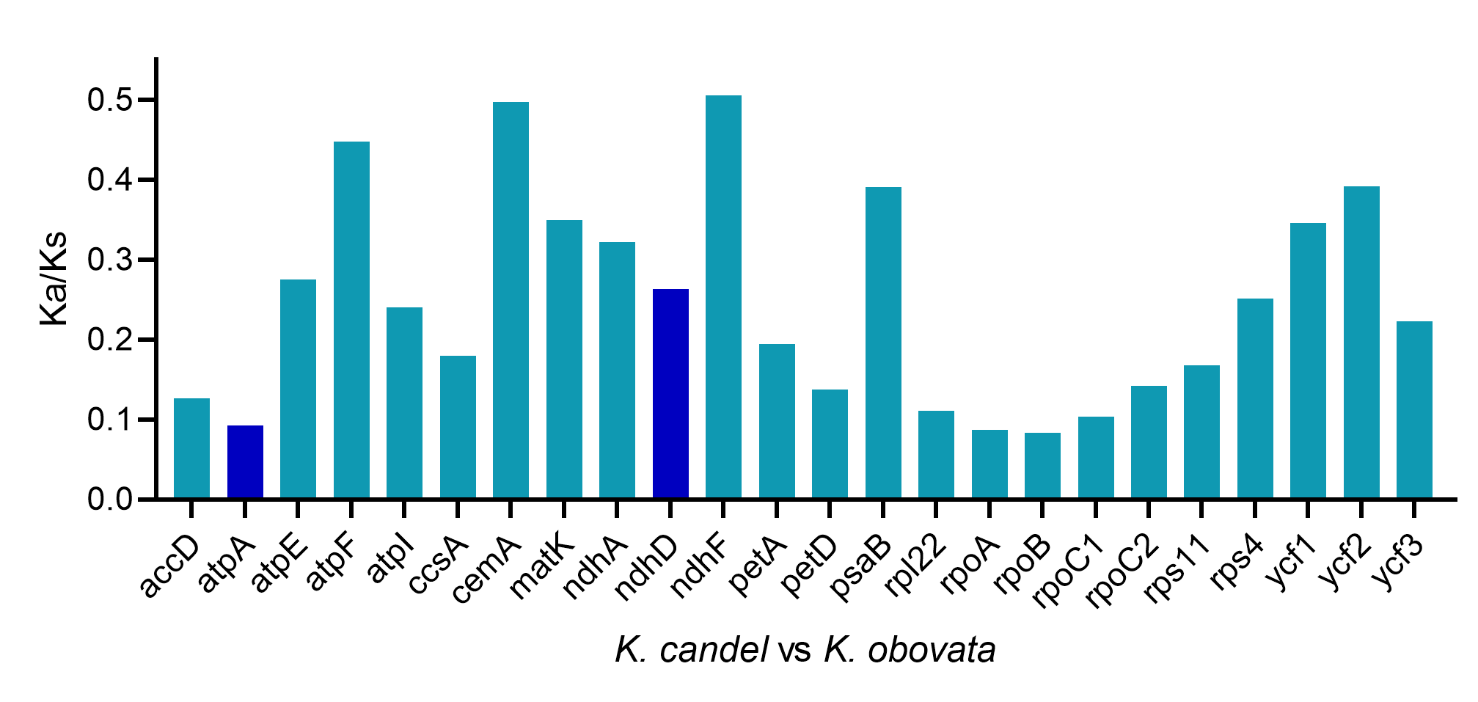


Figure S6. Ka/Ks ratios of 24 protein-coding genes for *K. candel* vs *K. obovata*.


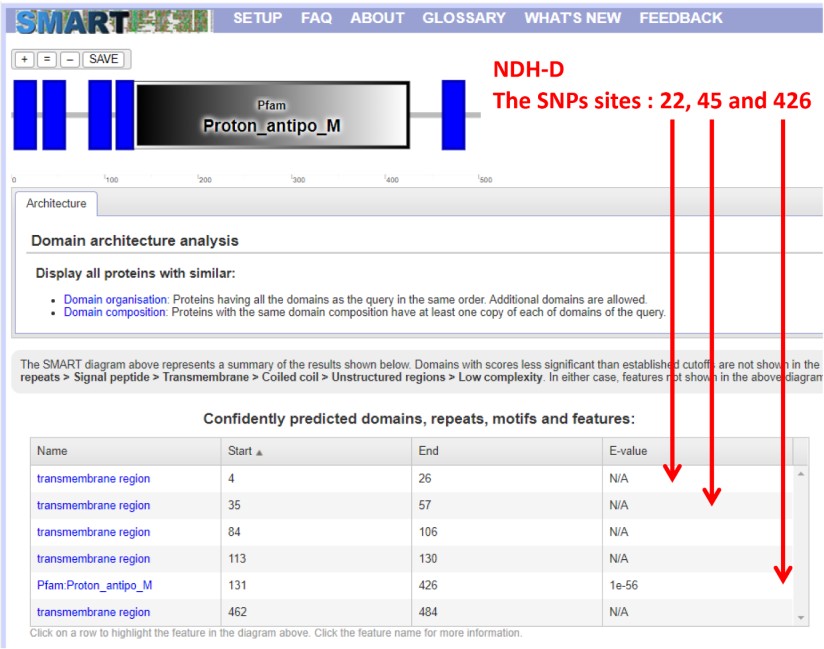
A

B


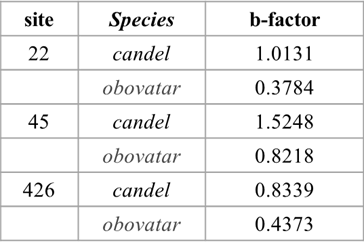


Figure S7. Prediction of the NDH-D protein domians using SMART showing the 22^nd^, 45^th^ and 426^th^ amino acids were located in the domain area(A), and b-factors values of these three amino acid sites (B).

A


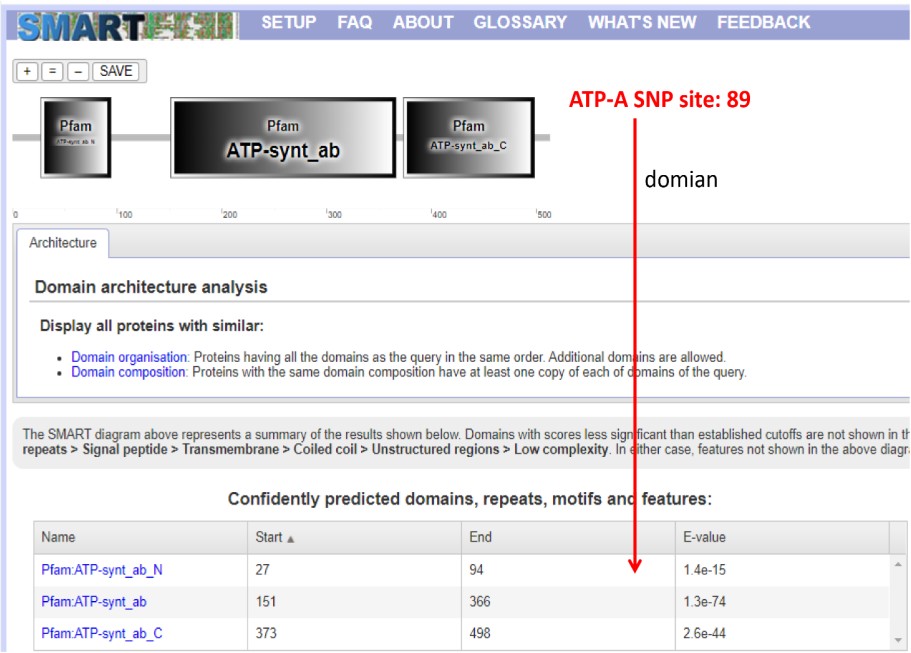


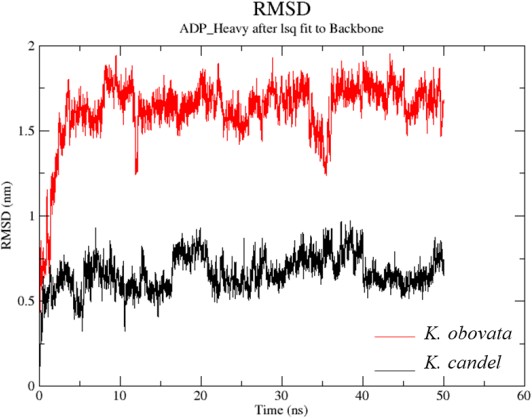

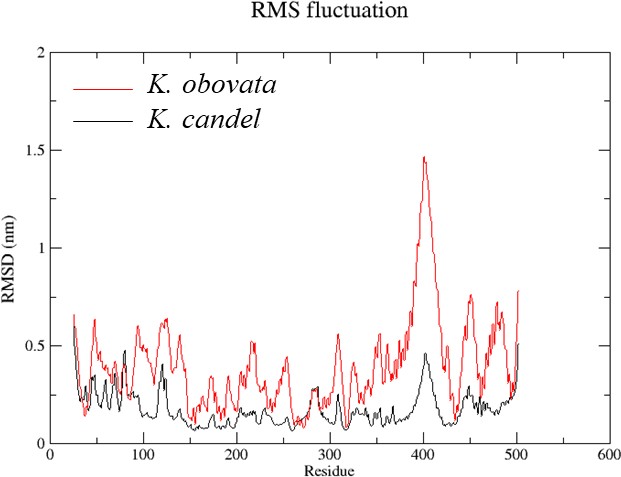
B C

Figure S8. The ATP-A protein domain predicted by using SMART showing the 89^th^ amino acid was located in domain area of the ATP-α subunit of the complex. (A) Evaluation of atpA and ligand ADP binding through molecular dynamic simulation by calculating the RMSD(B) and RMSfluctuation(C).


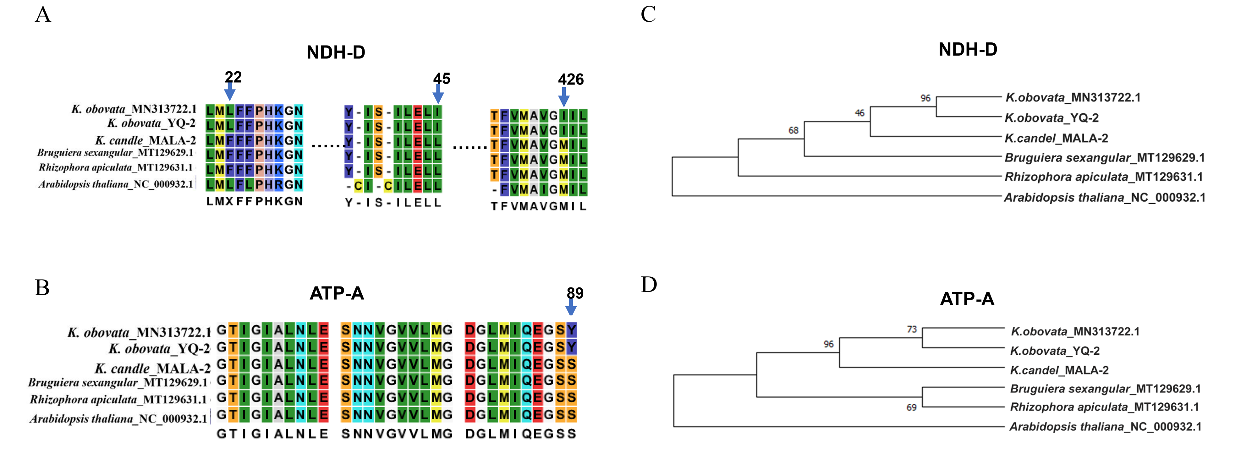


Figure S9. A. The NDH-D protein sequcing of *K. obovata*, *K. candel*, *Bruguicra sexangular*, *Rhizophora apiculata* and *Arabidopsis thaliana*. The blue arrow showed changes in 22 nd, 45 th and 426th loci distinguished by *K. candel* and *K. obovata*. B. The *atpA* protein sequences in 6 samples among four species. The blue arrow showed changes in 89 th loci. C. The phylogenetic tree based on NDH-D protein. D. The phylogenetic tree based on ATP-A protein.

**Supplymentary video**

The links to the animations showing the dynamics of ligand-protein binding during molecular dynamic simulations of ATP-A and

ADP in *K. candel* (A) and *K. obovata* (B).

atpA_ADP_can_movie (A)

atpA_ADP_obo_movie (B)
