# Supplementary material for: A comparison of 25 complete chloroplast genomes between sister mangrove species Kandelia obovata and Kandelia candel geographically separated by the South China Sea
Source: Front Plant Sci. 2023 Jan 4;13:1075353. doi: 10.3389/fpls.2022.1075353 (PMC9845719; doi:10.3389/fpls.2022.1075353)
Supplement: Supplementary file 2 [file DataSheet_2.docx]

| **Supplementary Table S1.** Collection sites of 25 samples of *Kandelia* | | | | | |
| --- | --- | --- | --- | --- | --- |
| Species | Country | Sample sites | Sample ID | longitude and latitude | GenBank Acc. |
| *K. obovata* | China | West Gate Island, Yandang Mountain Town, Yueqing City, Wenzhou City, Zhejiang Province | YQ-1 | 28°34'N,121°19'E | ON969326 |
| *K. obovata* | China | West Gate Island, Yandang Mountain Town, Yueqing City, Wenzhou City, Zhejiang Province | YQ-2 | 28°34'N,121°19'E | ON969327 |
| *K. obovata* | China | West Gate Island, Yandang Mountain Town, Yueqing City, Wenzhou City, Zhejiang Province | YQ-3 | 28°34'N,121°19'E | ON969328 |
| *K. obovata* | China | Xiaoyu, Qianqi Town, Fuding City (county-level city), Ningde City, Fujian Province | FD-1 | 27°29'N, 120°29'E | ON969314 |
| *K. obovata* | China | Jiulongjiang Estuary Mangrove Provincial Nature Reserve, Fujian/Jiulongjiang Estuary, Longhai  City, Zhangzhou City, Fujian Province | JLFG-1 | 24°26'N,117°54'E | ON969315 |
| *K. obovata* | China | Jiulongjiang Estuary Mangrove Provincial Nature Reserve, Fujian/Jiulongjiang Estuary, Longhai  City, Zhangzhou City, Fujian Province | JLFG-2 | 24°26'N,117°54'E | ON969316 |
| *K. obovata* | China | Jiulongjiang Estuary Mangrove Provincial Nature Reserve, Fujian/Jiulongjiang Estuary, Longhai  City, Zhangzhou City, Fujian Province | JLFG-3 | 24°26'N,117°54'E | ON969317 |
| *K. obovata* | China | Zhangjiang Estuary National Mangrove Wetland Nature Reserve, Yunxiao County, Zhangzhou  City, Fujian Province | YX-1 | 23°55'N,117°24'E | ON969329 |
| *K. obovata* | China | Zhangjiang Estuary National Mangrove Wetland Nature Reserve, Yunxiao County, Zhangzhou  City, Fujian Province | YX-2 | 23°55'N,117° 24'E | ON969330 |
| *K. obovata* | China | Zhangjiang Estuary National Mangrove Wetland Nature Reserve, Yunxiao County, Zhangzhou  City, Fujian Province | YX-3 | 23°55'N,117°24'E | ON969331 |
| *K. obovata* | China | Zhanjiang Mangrove National Nature Reserve, Leizhou Peninsula, Guangdong Province | LZ-1 | 21°37'N,109°47'E | ON969321 |
| *K. obovata* | China | Dongzhai Bay National Nature Reserve, Haikou City, Hainan Province | DZG-1 | 19°51'N,110°37'E | ON969310 |
| *K. obovata* | China | Xinying Mangrove National Wetland Park, Danzhou City, Hainan Province | DZXY-1 | 19°51'N,109°15'E | ON969311 |
| *K. obovata* | China | Xinying Mangrove National Wetland Park, Danzhou City, Hainan Province | DZXY-2 | 19°51'N,109°15'E | ON969312 |
| *K. obovata* | China | Xinying Mangrove National Wetland Park, Danzhou City, Hainan Province | DZXY-3 | 19°51'N,109 °15'E | ON969313 |
| *K. obovata* | China | Mangrove Wetland, Ledong County, Hainan Province | LD-1 | 18°45'N,109°10'E | ON969318 |
| *K. obovata* | China | Mangrove Wetland, Ledong County, Hainan Province | LD-2 | 18°45'N,109°10'E | ON969319 |
| *K. obovata* | China | Mangrove Wetland, Ledong County, Hainan Province | LD-3 | 18°45'N,109°10'E | ON969320 |
| *K. candel* | Thailand | Sai Daeng, Mueang Ranong District, Ranong 85130, Thailand | THAI | 10°10'N,98°43'E | ON969325 |
| *K. candel* | Malaysia | Muadzam Shah, Pahang, Malaysia | MALA-1 | 3°81'N,103°34' E | ON969322 |
| *K. candel* | Malaysia | Muadzam Shah, Pahang, Malaysia | MALA-2 | 3°81'N,103°35'E | ON969323 |
| *K. candel* | Malaysia | Muadzam Shah, Pahang, Malaysia | MALA-3 | 3°81'N,103°36'E | ON969324 |
| *K. candel* | Bangladesh | Sundarbans mangrove, Khulna region, Bangladesh | BGLA-1 | 22°16'N,89°26'E | ON969308 |
| *K. candel* | Bangladesh | Sundarbans mangrove, Khulna region, Bangladesh | BGLA-2 | 22°16'N,89°26'E | ON969310 |
| *K. candel* | Bangladesh | Sundarbans mangrove, Khulna region, Bangladesh | BGLA-3 | 22°16'N,89°26'E | ON969309 |

**Supplementary Table S2.** Gene contents in the chloroplast genomes of the 25 *Kandelia* cp genomes.

| **Category for genes** | **Group of genes** | **Name of genes** |
| --- | --- | --- |
| Photosynthesis related genes | Rubisco | *rbcL* |
|  | PhotosystemI | *psaA, psaB, psaC, psaI, psaJ* |
|  | Assembly/stability of photosystemI | *ycf3*, ycf4* |
|  | PhotosystemII | *psbA, psbB, psbC, psbD, psbE, psbF, psbJ,* ***psbK****, psbL, psbM, psbN,* ***psbI****, psbT, psbZ* |
|  | ATP synthase | *atpA, atpB, atpE, atpF*, atpH, atpI* |
|  | Cytochrome b/f compelx | *petA, petB*, petD*, petG, petL, petN* |
|  | Cytochrome C synthesis | *ccsA* |
|  | NADPH dehydrogenase | *ndhA*, ndhB*, ndhC, ndhD, ndhE, ndhG, ndhH, ndhI, ndhJ, ndhK* |
| Transcription and translation related genes | Transcription | *rpoA, rpoB, rpoC1*, rpoC2* |
|  | Ribosomal proteins | *rps11, rps12, rps14, rps15, rps18, rps19, rps2, rps3, rps4, rps7,*  *rps8, rpl14, rpl16, rpl2*, rpl20, rpl22, rpl23, rpl23, rpl33, rpl36* |
| RNA genes | Ribosomal RNA | *rrn16 rrn23 rrn4.5 rrn5* |
|  | Transfer RNA | *trnH-GUG,trnK-UUU*,****trnQ-UUG****, trnfM-CAU,trnG-UCC,*  *trnS-UGA,trnT-GGU,trnE-UUC,trnY-GUA,trnD-GUC,trnC-GCA,*  *trnR-UCU,trnG-UCC*,trnS-GCU,trnT-UGU,trnL-UAA*,trnF-GAA,*  ***trnS-GGA****, trnV-UAC*, trnM-CAU,trnW-CCA,trnP-UGG,trnI-CAU,*  *trnV-GAC,trnI-GAU*,trnA-UGC*,trnR-ACG,trnN-GUU,trnL-UAG,*  *trnL-CAA* |
| Other genes | RNA processing | *matK* |
|  | Carbon metabolism | *cemA* |
|  | Fatty acid synthesis | *accD* |
|  | Proteolysis | *clpP** |
| Genes of unknown function | Conserved reading frames | *ycf1, ycf2,* |

Intron-containing genes are marked by asterisks (*). The genes highlighted in bold were annotated in our study which were absent in the previous study (GenBank Acc. No NC042718, MN313722).

| **Supplemental Table S3.** The Ka/Ks value of homologous genes | | |  |  |  |  |
| --- | --- | --- | --- | --- | --- | --- |
| **Sequence** | **Method** | **Ka** | | **Ks** | **Ka/Ks** | |
| K102accD-K12accD | MA | 0.0023212 | | 0.0183923 | 0.126202 |  |
| **K102atpA-K12atpA** | **MA** | **0.001595** | | **0.017216** | **0.092647** |  |
| K102atpB-K12atpB | MA | NA | | NA | NA |  |
| K102atpE-K12atpE | MA | 0.0032297 | | 0.011727 | 0.275409 |  |
| K102atpF-K12atpF | MA | 0.004536 | | 0.0101277 | 0.447877 |  |
| K102atpH-K12atpH | MA | NA | | NA | NA |  |
| K102atpI-K12atpI | MA | 0.0017038 | | 0.00709 | 0.240309 |  |
| K102ccsA-K12ccsA | MA | 0.0023688 | | 0.0131759 | 0.179785 |  |
| K102cemA-K12cemA | MA | 0.0035501 | | 0.0071438 | 0.496945 |  |
| K102clpP-K12clpP | MA | NA | | NA | NA |  |
| K102matK-K12matK | MA | 0.0042851 | | 0.0122514 | 0.349763 |  |
| K102ndhA-K12ndhA | MA | 0.0033845 | | 0.0104995 | 0.322348 |  |
| K102ndhB-K12ndhB | MA | NA | | NA | NA |  |
| K102ndhC-K12ndhC | MA | NA | | NA | NA |  |
| **K102ndhD-K12ndhD** | **MA** | **0.003346** | | **0.012712** | **0.263224** |  |
| K102ndhE-K12ndhE | MA | NA | | NA | NA |  |
| K102ndhF-K12ndhF | MA | 0.0050351 | | 0.0099675 | 0.505151 |  |
| K102ndhG-K12ndhG | MA | 7.96E-06 | | 0.0079619 | 0.001 |  |
| K102ndhH-K12ndhH | MA | 1.60E-05 | | 0.016011 | 0.001 |  |
| K102ndhI-K12ndhI | MA | 2.15E-05 | | 0.0214749 | 0.001 |  |
| K102ndhJ-K12ndhJ | MA | NA | | NA | NA |  |
| K102ndhK-K12ndhK | MA | NA | | NA | NA |  |
| K102petA-K12petA | MA | 0.0041086 | | 0.0211467 | 0.194289 |  |
| K102petB-K12petB | MA | 6.63E-06 | | 0.0066296 | 0.001 |  |
| K102petD-K12petD | MA | 0.0023918 | | 0.017443 | 0.137118 |  |
| K102petG-K12petG | MA | NA | | NA | NA |  |
| K102petL-K12petL | MA | NA | | NA | NA |  |
| K102petN-K12petN | MA | NA | | NA | NA |  |
| K102psaA-K12psaA | MA | 1.96E-06 | | 0.0019591 | 0.001 |  |
| K102psaB-K12psaB | MA | 0.0013559 | | 0.0034671 | 0.391085 |  |
| K102psaC-K12psaC | MA | NA | | NA | NA |  |
| K102psaI-K12psaI | MA | NA | | NA | NA |  |
| K102psbA-K12psbA | MA | 1.45E-05 | | 0.0144595 | 0.001 |  |
| K102psbB-K12psbB | MA | 8.39E-06 | | 0.0083935 | 0.001 |  |
| K102psbC-K12psbC | MA | 7.80E-06 | | 0.0077966 | 0.001 |  |
| K102psbD-K12psbD | MA | 3.45E-06 | | 0.003447 | 0.001 |  |
| K102psbE-K12psbE | MA | 2.08E-05 | | 0.020755 | 0.001 |  |
| K102psbF-K12psbF | MA | NA | | NA | NA |  |
| K102psbH-K12psbH | MA | 2.42E-05 | | 0.0242109 | 0.001 |  |
| K102psbI-K12psbI | MA | NA | | NA | NA |  |
| K102psbK-K12psbK | MA | NA | | NA | NA |  |
| K102psbL-K12psbL | MA | NA | | NA | NA |  |
| K102psbM-K12psbM | MA | NA | | NA | NA |  |
| K102psbN-K12psbN | MA | NA | | NA | NA |  |
| K102psbT-K12psbT | MA | NA | | NA | NA |  |
| K102psbZ-K12psbZ | MA | 2.20E-05 | | 0.0220188 | 0.001 |  |
| K102rbcL-K12rbcL | MA | NA | | NA | NA |  |
| K102rpl14-K12rpl14 | MA | 6.22E-05 | | 0.0621814 | 0.001 |  |
| K102rpl16-K12rpl16 | MA | 0.0128584 | | 0.0002572 | NA |  |
| K102rpl20-K12rpl20 | MA | 0.0168446 | | 0.0003369 | NA |  |
| K102rpl22-K12rpl22 | MA | 0.0027547 | | 0.0247691 | 0.111215 |  |
| K102rpl23-K12rpl23 | MA | NA | | NA | NA |  |
| K102rpl33-K12rpl33 | MA | 2.25E-05 | | 0.0225276 | 0.001 |  |
| K102rpl36-K12rpl36 | MA | 4.19E-05 | | 0.0418883 | 0.001 |  |
| K102rpoA-K12rpoA | MA | 0.0013642 | | 0.0156336 | 0.0872609 |  |
| K102rpoB-K12rpoB | MA | 0.0007513 | | 0.0089988 | 0.083493 |  |
| K102rpoC1-K12rpoC1 | MA | 0.0010681 | | 0.0103105 | 0.103592 |  |
| K102rpoC2-K12rpoC2 | MA | 0.0013594 | | 0.0095458 | 0.142408 |  |
| K102rps11-K12rps11 | MA | 0.0033254 | | 0.019864 | 0.167408 |  |
| K102rps12-K12rps12 | MA | 0.0068361 | | 0.0001377 | NA |  |
| K102rps14-K12rps14 | MA | 1.43E-05 | | 0.0143057 | 0.001 |  |
| K102rps15-K12rps15 | MA | NA | | NA | NA |  |
| K102rps18-K12rps18 | MA | NA | | NA | NA |  |
| K102rps19-K12rps19 | MA | NA | | NA | NA |  |
| K102rps2-K12rps2 | MA | NA | | NA | NA |  |
| K102rps3-K12rps3 | MA | 1.15E-05 | | 0.0115204 | 0.001 |  |
| **K102rps4-K12rps4** | **MA** | **0.004613** | | **0.018334** | **0.251611** |  |
| K102rps7-K12rps7 | MA | NA | | NA | NA |  |
| K102rps8-K12rps8 | MA | 5.11E-05 | | 0.051096 | 0.001 |  |
| K102ycf1-K12ycf1 | MA | 0.0049994 | | 0.0144287 | 0.34649 |  |
| K102ycf2-K12ycf2 | MA | 0.000536 | | 0.0013665 | 0.3922 |  |
| K102ycf3-K12ycf3 | MA | 0.0025817 | | 0.0115529 | 0.223465 |  |
| K102ycf4-K12ycf4 | MA | 7.60E-06 | | 0.0075999 | 0.001 |  |

| **Supplementary Table S4.** The number and distribution of SSRs | | | | | | | | | | | | | | | | | | | | | | |
| --- | --- | --- | --- | --- | --- | --- | --- | --- | --- | --- | --- | --- | --- | --- | --- | --- | --- | --- | --- | --- | --- | --- |
| ID | The numbe r of Mono- | Percentage of the total SSRs (%) | The number of di- | Percenta ge of the total SSRs (%) | The number of tri- | Percentage of the total SSRs (%) | AT content (%) | GC content (%) | LSC | | | IRb | | | SSC | | | IRa | | | Total |  |
|  |  |  |  |  |  |  |  |  |  |  |  |  |  |  |  |  |  |  |  |  |  |  |
|  |  |  |  |  |  |  |  |  |  |  |  |  |  |  |  |  |  |  |  |  |  |  |
|  |  |  |  |  |  |  |  |  | Mono- | Di- | Tri- | Mono- | Di- | Tri- | Mono- | Di- | Tri- | Mono- | Di- | Tri- |  |  |
| DZG | 71 | 92.21 | 5 | 6.49 | 1 | 0.01 | 1.3 | 0 | 51 | 5 | 1 | 4 | 0 | 0 | 12 | 0 | 0 | 4 | 0 | 0 | 77 |  |
| FD | 68 | 93.15 | 4 | 5.48 | 1 | 0.01 | 1.37 | 0 | 50 | 4 | 1 | 4 | 0 | 0 | 10 | 0 | 0 | 4 | 0 | 0 | 73 |  |
| JLFG-1 | 69 | 92 | 5 | 6.67 | 1 | 0.01 | 1.33 | 0 | 49 | 5 | 1 | 4 | 0 | 0 | 11 | 0 | 0 | 5 | 0 | 0 | 75 |  |
| JLFG-2 | 69 | 92 | 5 | 6.67 | 1 | 0.01 | 1.33 | 0 | 49 | 5 | 1 | 4 | 0 | 0 | 11 | 0 | 0 | 5 | 0 | 0 | 75 |  |
| JLFG-3 | 69 | 92 | 5 | 6.67 | 1 | 0.01 | 1.33 | 0 | 49 | 5 | 1 | 4 | 0 | 0 | 11 | 0 | 0 | 5 | 0 | 0 | 75 |  |
| LD-1 | 71 | 92.21 | 5 | 6.49 | 1 | 0.01 | 1.3 | 0 | 51 | 5 | 1 | 4 | 0 | 0 | 12 | 0 | 0 | 4 | 0 | 0 | 77 |  |
| LD-2 | 71 | 92.21 | 5 | 6.49 | 1 | 0.01 | 1.3 | 0 | 51 | 5 | 1 | 4 | 0 | 0 | 11 | 0 | 0 | 5 | 0 | 0 | 77 |  |
| LD-3 | 71 | 92.21 | 5 | 6.49 | 1 | 0.01 | 1.3 | 0 | 51 | 5 | 1 | 4 | 0 | 0 | 11 | 0 | 0 | 5 | 0 | 0 | 77 |  |
| DZXY-1 | 71 | 91.03 | 6 | 7.69 | 1 | 0.01 | 1.28 | 0 | 51 | 5 | 1 | 3 | 1 | 0 | 12 | 0 | 0 | 5 | 0 | 0 | 78 |  |
| DZXY-2 | 71 | 91.03 | 6 | 7.69 | 1 | 0.01 | 1.28 | 0 | 51 | 5 | 1 | 3 | 1 | 0 | 12 | 0 | 0 | 5 | 0 | 0 | 78 |  |
| DZXY-3 | 71 | 91.03 | 6 | 7.69 | 1 | 0.01 | 1.28 | 0 | 51 | 5 | 1 | 3 | 1 | 0 | 12 | 0 | 0 | 5 | 0 | 0 | 78 |  |
| YQ-1 | 69 | 92 | 5 | 6.67 | 1 | 0.01 | 1.33 | 0 | 51 | 5 | 1 | 4 | 0 | 0 | 9 | 0 | 0 | 5 | 0 | 0 | 75 |  |
| YQ-2 | 69 | 92 | 5 | 6.67 | 1 | 0.01 | 1.33 | 0 | 51 | 5 | 1 | 4 | 0 | 0 | 9 | 0 | 0 | 5 | 0 | 0 | 75 |  |
| YQ-3 | 71 | 92.21 | 5 | 6.49 | 1 | 0.01 | 1.3 | 0 | 51 | 5 | 1 | 4 | 0 | 0 | 11 | 0 | 0 | 5 | 0 | 0 | 77 |  |
| LZ | 66 | 90.41 | 6 | 8.22 | 1 | 0.01 | 1.37 | 0 | 47 | 6 | 1 | 4 | 0 | 0 | 10 | 0 | 0 | 5 | 0 | 0 | 73 |  |
| YX-1 | 67 | 91.78 | 5 | 6.85 | 1 | 0.01 | 1.37 | 0 | 47 | 5 | 1 | 4 | 0 | 0 | 11 | 0 | 0 | 5 | 0 | 0 | 73 |  |
| YX-2 | 69 | 92 | 5 | 6.67 | 1 | 0.01 | 1.33 | 0 | 49 | 5 | 1 | 4 | 0 | 0 | 11 | 0 | 0 | 5 | 0 | 0 | 75 |  |
| YX-3 | 69 | 92 | 5 | 6.67 | 1 | 0.01 | 1.33 | 0 | 49 | 5 | 1 | 4 | 0 | 0 | 10 | 0 | 0 | 6 | 0 | 0 | 75 |  |
| BGLA-1 | 66 | 90.41 | 6 | 8.22 | 1 | 0.01 | 1.37 | 1.37 | 50 | 5 | 1 | 4 | 0 | 0 | 7 | 1 | 0 | 5 | 0 | 0 | 73 |  |
| BGLA-2 | 66 | 90.41 | 6 | 8.22 | 1 | 0.01 | 1.37 | 1.37 | 50 | 5 | 1 | 4 | 0 | 0 | 7 | 1 | 0 | 5 | 0 | 0 | 73 |  |
| BGLA-3 | 75 | 93.75 | 4 | 5 | 1 | 0.01 | 1.25 | 98.75 | 57 | 3 | 1 | 4 | 0 | 0 | 9 | 1 | 0 | 6 | 0 | 0 | 80 |  |
| MALA-1 | 75 | 93.75 | 4 | 5 | 1 | 0.01 | 1.25 | 98.75 | 57 | 3 | 1 | 4 | 0 | 0 | 9 | 1 | 0 | 5 | 0 | 0 | 80 |  |
| MALA-2 | 76 | 93.83 | 4 | 4.94 | 1 | 0.01 | 1.23 | 98.77 | 57 | 3 | 1 | 5 | 0 | 0 | 10 | 1 | 0 | 4 | 0 | 0 | 81 |  |
| MALA-3 | 75 | 0.94 | 4 | 5 | 1 | 0.01 | 1.25 | 98.75 | 57 | 3 | 1 | 4 | 0 | 0 | 9 | 1 | 0 | 5 | 0 | 0 | 80 |  |
| THAI | 66 | 90.41 | 6 | 8.22 | 1 | 0.01 | 1.37 | 98.63 | 50 | 5 | 1 | 4 | 0 | 0 | 7 | 1 | 0 | 5 | 0 | 0 | 73 |  |

**Supplementary Table S5** Primers information of sugarcane SSR with polymorphic amplification

| Sequence ID | Primer ID | Primer Sequence | Primer Tm | Primer GC | Product Size | Product Tm |
| --- | --- | --- | --- | --- | --- | --- |
| ZXY10-2_644_647 | ZXY10-2_644_647_1_F | AGCTTGGTATTGCTCCCCT | 58.285 | 52.632 | 107 | 78.6 |
| ZXY10-2_644_647 | ZXY10-2_644_647_1_R | AGACCTAGCTGCTGTCGA | 57.255 | 55.556 | 107 | 78.6 |
| ZXY10-2_7794_7794 | ZXY10-2_7794_7794_1_F | GCCCCGAAACCGAGTGAA | 59.969 | 61.111 | 250 | 79.5 |
| ZXY10-2_7794_7794 | ZXY10-2_7794_7794_1_R | CGGAATCGCTGCTTTCGC | 59.978 | 61.111 | 250 | 79.5 |
| ZXY10-2_21052_21053 | ZXY10-2_21052_21053_1_F | TCGTGTACATTGCATTCCCCT | 59.719 | 47.619 | 273 | 79.2 |
| ZXY10-2_21052_21053 | ZXY10-2_21052_21053_1_R | TGGGCGAAGAAAGTCTGC | 57.621 | 55.556 | 273 | 79.2 |
| ZXY10-2_21054_21055 | ZXY10-2_21054_21055_1_F | TCGTGTACATTGCATTCCCCT | 59.719 | 47.619 | 273 | 79.2 |
| ZXY10-2_21054_21055 | ZXY10-2_21054_21055_1_R | TGGGCGAAGAAAGTCTGC | 57.621 | 55.556 | 273 | 79.2 |
| ZXY10-2_43629_43633 | ZXY10-2_43629_43633_1_F | GCGGGCCAAGCTGTAGAA | 60.046 | 61.111 | 210 | 81.4 |
| ZXY10-2_43629_43633 | ZXY10-2_43629_43633_1_R | GCAAGCAGCAAATCCAAGGT | 59.681 | 50 | 210 | 81.4 |
| ZXY10-2_43698_43699 | ZXY10-2_43698_43699_1_F | ACCTTGGATTTGCTGCTTGC | 59.681 | 50 | 199 | 77.8 |
| ZXY10-2_43698_43699 | ZXY10-2_43698_43699_1_R | GGGGGAAGGAAGAAAGCGA | 59.317 | 57.895 | 199 | 77.8 |
| ZXY10-2_43711_43713 | ZXY10-2_43711_43713_1_F | ACCTTGGATTTGCTGCTTGC | 59.681 | 50 | 199 | 77.8 |
| ZXY10-2_43711_43713 | ZXY10-2_43711_43713_1_R | GGGGGAAGGAAGAAAGCGA | 59.317 | 57.895 | 199 | 77.8 |
| ZXY10-2_45515_45515 | ZXY10-2_45515_45515_1_F | ACGAGTTTTCCAACAAGCCT | 57.947 | 45 | 180 | 81.5 |
| ZXY10-2_45515_45515 | ZXY10-2_45515_45515_1_R | AACTCCCCTCCTTCCCCC | 59.872 | 66.667 | 180 | 81.5 |
| ZXY10-2_45518_45518 | ZXY10-2_45518_45518_1_F | ACGAGTTTTCCAACAAGCCT | 57.947 | 45 | 180 | 81.5 |
| ZXY10-2_45518_45518 | ZXY10-2_45518_45518_1_R | AACTCCCCTCCTTCCCCC | 59.872 | 66.667 | 180 | 81.5 |
| ZXY10-2_51911_51912 | ZXY10-2_51911_51912_1_F | GCCCGTTGGAAAAATGGGG | 59.705 | 57.895 | 173 | 80.9 |
| ZXY10-2_51911_51912 | ZXY10-2_51911_51912_1_R | GCCCGCTTAGCTCAGAGG | 59.892 | 66.667 | 173 | 80.9 |
| ZXY10-2_67133_67135 | ZXY10-2_67133_67135_1_F | ACCCAAGAGTCCTCTTTTGTT | 57.046 | 42.857 | 241 | 76.7 |
| ZXY10-2_67133_67135 | ZXY10-2_67133_67135_1_R | CTTTGCGGCGAATGTCCA | 58.737 | 55.556 | 241 | 76.7 |
| ZXY10-2_67160_67161 | ZXY10-2_67160_67161_1_F | ACCCAAGAGTCCTCTTTTGTT | 57.046 | 42.857 | 241 | 76.7 |
| ZXY10-2_67160_67161 | ZXY10-2_67160_67161_1_R | CTTTGCGGCGAATGTCCA | 58.737 | 55.556 | 241 | 76.7 |
| ZXY10-2_67175_67176 | ZXY10-2_67175_67176_1_F | ACCCAAGAGTCCTCTTTTGTT | 57.046 | 42.857 | 241 | 76.7 |
| ZXY10-2_67175_67176 | ZXY10-2_67175_67176_1_R | CTTTGCGGCGAATGTCCA | 58.737 | 55.556 | 241 | 76.7 |
| ZXY10-2_67176_67177 | ZXY10-2_67176_67177_1_F | ACCCAAGAGTCCTCTTTTGTT | 57.046 | 42.857 | 241 | 76.7 |
| ZXY10-2_67176_67177 | ZXY10-2_67176_67177_1_R | CTTTGCGGCGAATGTCCA | 58.737 | 55.556 | 241 | 76.7 |
| ZXY10-2_69612_69613 | ZXY10-2_69612_69613_1_F | TGGGGAATTACTTGTACCGCA | 59.371 | 47.619 | 240 | 79.5 |
| ZXY10-2_69612_69613 | ZXY10-2_69612_69613_1_R | TCTCCGCATTTAGCCTTTGC | 58.905 | 50 | 240 | 79.5 |
| ZXY10-2_73830_73831 | ZXY10-2_73830_73831_1_F | CAGCTTATCTTGCCAGTTCGG | 59.332 | 52.381 | 243 | 74.4 |
| ZXY10-2_73830_73831 | ZXY10-2_73830_73831_1_R | TCCCTTTCCGCCCGAGTA | 59.962 | 61.111 | 243 | 74.4 |
| ZXY10-2_73842_73843 | ZXY10-2_73842_73843_1_F | CAGCTTATCTTGCCAGTTCGG | 59.332 | 52.381 | 243 | 74.4 |
| ZXY10-2_73842_73843 | ZXY10-2_73842_73843_1_R | TCCCTTTCCGCCCGAGTA | 59.962 | 61.111 | 243 | 74.4 |
| ZXY10-2_73940_73941 | ZXY10-2_73940_73941_1_F | TACTCGGGCGGAAAGGGA | 59.962 | 61.111 | 155 | 76.2 |
| ZXY10-2_73940_73941 | ZXY10-2_73940_73941_1_R | TCGTTAACGGCCACTCGT | 58.958 | 55.556 | 155 | 76.2 |
| ZXY10-2_76310_76311 | ZXY10-2_76310_76311_1_F | ACCTTCCCAACCCCGATT | 58.068 | 55.556 | 211 | 80 |
| ZXY10-2_76310_76311 | ZXY10-2_76310_76311_1_R | AATGAAGGACGGGGCTCC | 59.001 | 61.111 | 211 | 80 |
| ZXY10-2_77861_77866 | ZXY10-2_77861_77866_1_F | TCGGCGATTTAGCAATCCCA | 59.821 | 50 | 170 | 75.5 |
| ZXY10-2_77861_77866 | ZXY10-2_77861_77866_1_R | GGGGGCCCATTTCAGCAT | 60.042 | 61.111 | 170 | 75.5 |
| ZXY10-2_87395_87396 | ZXY10-2_87395_87396_1_F | TCATTCCTGCGTGAACCCA | 59.242 | 52.632 | 234 | 80.4 |
| ZXY10-2_87395_87396 | ZXY10-2_87395_87396_1_R | TCGACGGGGGCGAATTTT | 59.652 | 55.556 | 234 | 80.4 |
| ZXY10-2_119836_119837 | ZXY10-2_119836_119837_1_F | TGTCGTTTTCCAAGAAACCCT | 57.993 | 42.857 | 229 | 76.9 |
| ZXY10-2_119836_119837 | ZXY10-2_119836_119837_1_R | AGCTTGTTTTACAGCCGGA | 57.282 | 47.368 | 229 | 76.9 |
| ZXY10-2_130118_130118 | ZXY10-2_130118_130118_1_F | ATGTACCTTGCAGCCCGT | 58.922 | 55.556 | 202 | 77.3 |
| ZXY10-2_130118_130118 | ZXY10-2_130118_130118_1_R | ACAACCTCTTCCCAACTCTTT | 57.046 | 42.857 | 202 | 77.3 |
